# A Thorough Characterization of the Tellurocyanate Anion

**DOI:** 10.1002/anie.202507543

**Published:** 2025-06-03

**Authors:** Hennes Günther, Florian Weigend, Xiulan Xie, Wenjin Cao, Xiao‐Fei Gao, Xue‐Bin Wang, Frank Tambornino

**Affiliations:** ^1^ Department of Chemistry Philipps University of Marburg Hans‐Meerwein‐Str. 4 D‐35043 Marburg Germany; ^2^ Institute for Quantum Materials and Technologies Karlsruhe Institute of Technology Hermann‐von‐Helmholtz‐Platz 1 D‐76344 Eggenstein‐Leopoldshafen Germany; ^3^ Physical Sciences Division Pacific Northwest National Laboratory Richland Washington 99352 USA

**Keywords:** Bonding analysis, Cyanates, DFT, Negative ion photoelectron spectroscopy, Nuclear magnetic resonance

## Abstract

Tellurocyanate, [TeCN]^−^, is the heaviest group 16 congener of the cyanate anion, [OCN]^−^. Due to the relative instability of the C─Te bond, tellurocyanate chemistry has seen only scarce attention. Here, we present the facile synthesis and thorough characterization of [K@crypt‐222][TeCN]. The anion is essentially linear with interatomic distances C─N = 1.150(6)Å and C─Te = 2.051(4)Å, thus approximating a C≡N triple bond and for C─Te a bond order between 1 and 2. Fully ^13^C and ^15^N labeled [Te^13^C^15^N]^−^ allowed for the extraction of chemical shifts and all possible coupling constants (^13^C = 77.8 ppm, ^15^N = 285.7 ppm, ^125^Te = −566 ppm, ^1^ *J*_13C‐15N_ = 8 Hz, ^1^ *J*_13C‐125Te_ = 748 Hz, ^2^ *J*_15N‐125Te_ = 55 Hz), which were also determined independently by quantum chemical calculations. In the series [*Ch*CN]^−^ (*Ch* = O─Te), [TeCN]^−^ shows the strongest spin‐orbit coupling (SOC) induced heavy‐atom effect on the light‐atom shielding (SO‐HALA‐effect). In contrast, ^15^N shifts are also well described without considering relativistic effects and/or SOC. Negative‐ion photoelectron spectroscopy was used to extract the electron affinity (EA = 3.034 eV) and spin‐orbit splitting (3807 cm^−1^) of [TeCN]^•^. These values continue the trends of falling EA and rising SOC in the series [*Ch*CN]^•^.

## Graphical abstract

[K@crypt‐222][TeCN] was synthesized from KCN, Te, and crypt‐222 in acetone. We discuss fundamental properties of [TeCN]^−^: Single crystal X‐ray diffraction, nuclear magnetic resonance (^13^C, ^15^N, ^125^Te), vibrational spectroscopy, and negative ion photoelectron spectroscopy. All experimental data is supported by high‐level quantum‐chemical calculations.

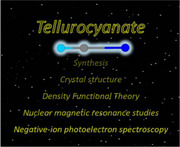

## Introduction

The year 1925 saw the introduction of the pseudohalogen concept.^[^ ^1^ ^]^ In their seminal paper, the authors mainly discuss the cyanate anion and its chalcogen congeners with respect to their similarity to group 17 ions/elements. They focused their discussion on cyanate, [OCN]^−^, thiocyanate, [SCN]^−^, selenocyanate, [SeCN]^−^, and tellurocyanate, [TeCN]^−^, which today count as archetypical pseudohalogen ions and feature prominently in chemistry textbooks. More recently, heavy group 15 congeners have been synthesized, ^[^ ^2^, ^3^, ^4^, ^5^ ^]^ and especially the chemistry of the phosphaethynolate anion, [OCP]^−^, has seen widespread use.^[^ ^6^ ^]^

Although the chemistry of cyanates and thiocyanates has been extensively studied, selenocyanates and especially tellurocyanates remain rather unexplored. [TeCN]^−^ has perhaps been synthesized as early as 1925, however, it was not isolated, and its synthesis was disputed.^[^ ^1^, ^2^, ^3^, ^4^, ^5^, ^6^, ^7^ ^]^ Its first spectroscopic characterization (IR spectroscopy, as its tetraethylammonium salt) was performed almost half a decade later in 1968.^[^ ^8^ ^]^ The unambiguous crystallographic characterization was achieved in 1979, where the structure of [PPN][TeCN], first synthesized in 1977, was elucidated.^[^ ^9^, ^10^ ^]^ Due to limitations of that time, no absorption correction was performed, and the refinement included solely isotropic carbon atoms, leading to somewhat unreliable interatomic distances. Since then, the only other published structure comprising the [TeCN]^−^ anion is [K@18‐crown‐6][TeCN].^[^ ^11^ ^]^ However, here the [TeCN]^−^ is bridging two cations and its synthesis was serendipitous. Due to the relatively weak C─Te bond, it does not come as a surprise that the chemistry of [TeCN]^−^ is quite unexplored. Even key spectroscopic signatures, e.g., data on NMR spectra, is absent in the literature.

In this work, we present the facile synthesis of [K@crypt‐222][TeCN] by reaction of potassium cyanide, elemental tellurium, and crypt‐222 in acetone. [K@crypt‐222][TeCN] is a colorless crystalline solid that is sensitive to light and air, readily releasing tellurium upon exposure. We disclose a full characterization by single crystal X‐ray diffraction, NMR, IR, and Raman spectroscopy. Using ^13^C and ^15^N labeling, we prepared [K@crypt‐222][Te^13^C^15^N], which allowed us to get full insight into the NMR spectroscopic properties and thus the underlying electronic structure. Density functional theory corroborates our findings and allows for a deeper insight into the electronic structure of [TeCN]^−^. Negative‐ion photoelectron spectroscopy (NIPES) was employed to gain further insight into electron affinity (EA) and spin‐orbit splitting of the neutral [TeCN]^•^ molecule.

## Results and Discussion

### Synthesis and Crystal Structure

**Figure 1:**
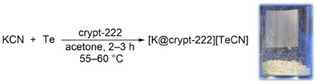
Synthesis of [K@crypt‐222][TeCN] (left). Bulk sample of [K@crypt‐222][TeCN] (right).

[K@crypt‐222][TeCN] was synthesized by the reaction of potassium cyanide with elemental tellurium and crypt‐222 in boiling acetone or at 55–60°C in a closed system (Figure 1).

**Figure 2:**
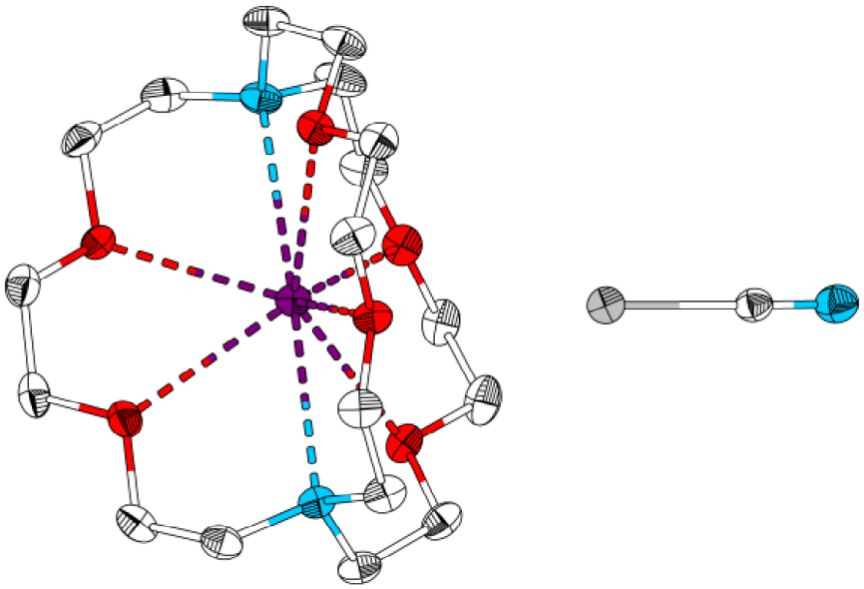
Molecular structure of [K@crypt‐222][TeCN] in the single crystal drawn with 50% displacement ellipsoids at 100K. Hydrogen atoms are omitted for clarity. Color code: C white, N blue, O red, K purple, and Te grey.

The reaction progress can be followed by observing the steady decrease of elemental tellurium in the reaction mixture, which turns colorless/off‐white when all tellurium is consumed. Depending on the concentration of [K@crypt‐222][TeCN], small amounts precipitate as colorless solid as the reaction mixture is cooled to room temperature. After filtration, a slightly yellow solution of [K@crypt‐222][TeCN] in acetone is obtained. Regardless of the concentration, solutions of [K@crypt‐222][TeCN] in acetone are highly sensitive towards air and moderately sensitive towards light. Contact with protic solvents, e.g., water or alcohols, leads to deposition of elemental tellurium either immediately or within seconds, respectively. Isolation of [K@crypt‐222][TeCN] proved to be difficult, as the success of the isolation is strongly dependent on the initial concentration of the solution of [K@crypt‐222][TeCN] in acetone. For example, concentrated solutions release significant amounts of elemental tellurium by adding antisolvents like *n*‐hexane, quickly removing acetone in vacuo, or dilution with acetone. In contrast, dilute solutions are comparatively stable; their dilution or the addition of antisolvents does not lead to decomposition. If acetone is removed in vacuo, it is necessary that the solvent evaporation is performed slowly. If an equivalent reaction is carried out without crypt‐222 in the reaction mixture, no reaction can be observed. It is reported that analogous reactions of K[CN] with tellurium were successful in boiling dimethyl sulfoxide (DMSO), yielding dissolved K[TeCN]. However, this reaction approach is not suitable for isolating solvent free K[TeCN].^[^ ^12^ ^]^ To obtain single crystals suitable for single crystals X‐ray diffraction, an acetone solution of [K@crypt‐222][TeCN] was layered with *n*‐hexane. In addition to a precipitate of dark grey tellurium (Figure S16), colorless crystals of [K@crypt‐222][TeCN] are obtained after three days. [K@crypt‐222][TeCN] crystallizes in the monoclinic crystal system in space group *P*2_1_/*c* (No. 14, *a* = 11.7362(5) Å, *b* = 14.3520(5) Å, *c* = 14.9300(7) Å, *β* = 92.161(4)°) with one independent unit in the asymmetric unit and four units in the unit cell (*Z* = 4, see Figure 2, for additional information see Table S1).

In the crystal structure, [K@crypt‐222]^+^ and [TeCN]^−^ ions are separated and do not show direct interaction through coordination. This is similar to the structure of [PPN][TeCN] but differs from the structure of [K@18‐crown‐6][TeCN], where the [TeCN]^−^ anion is wedged in between two [K@18‐crown‐6]^+^ cations. Akin to its respective cyanate congeners, the [TeCN]^−^anion is essentially linear. With ∢_(Te─C─N) _= 179.2(4)°, the angle is similar to the respective angle in [K@18‐crown‐6][TeCN] (179.6(5)°). In contrast, it differs significantly from the respective angle in [PPN][TeCN], which is 174.843(4)°; however, the authors state that the data was not absorption corrected and might not be entirely reliable.^[^ ^10^, ^11^ ^]^ The C─N bond length is 1.150(6) Å and is in the range of a C≡N triple bond (1.14 Å).^[^ ^13^ ^]^ With 2.051(4) Å, the C─Te bond shows contributions of both single (2.11 Å) and double bond (1.95 Å) character.^[^ ^13^ ^]^ In comparison to the C─Te bond of the previously published dimethyl telluride (2.131(6)–2.143(6) Å), which approximates a pure single bond, it is approximately 0.08 Å shorter.^[^ ^14^ ^]^ A comparison of bond lengths with [K@(18‐crown‐6)][TeCN] (C─N: 1.148(6) Å, C─Te: 2.031(5) Å) matches well, although in this crystal structure the tellurocyanate anion acts as a bridging ligand between [K@18‐crown‐6]^+^ units. Bond lengths determined by us are somewhat different than their counterparts in [PPN][TeCN] (C─N: 1.07(1) Å, C─Te: 2.02(1)–Å), which is most likely an effect of the aforementioned missing absorption correction. Since in literature no single crystal data with general composition [K@crypt‐222][*Ch*CN] (*Ch* = O, S, Se) have been described, we chose compounds for bond lengths comparison in which the respective chalcogenide cyanate anion is only exposed to weak interaction with the respective cation (Table S2).^[^ ^15^, ^16^, ^17^ ^]^ In all [*Ch*CN]^−^ anions (*Ch *= O‐Te), the C─N bond length is in the range of 1.145(4)–1.161(9) Å, indicating slightly elongated C≡N triple bonds (1.14 Å). In contrast, the C─*Ch* bond lengths increase with increasing period of the chalcogen atom from C─O (1.245(4) Å) to C─Te (2.051(4) Å). The *Ch*─C─N angle is nearly 180° in all discussed compounds.

### Chemical Bonding Analysis

**Figure 3:**
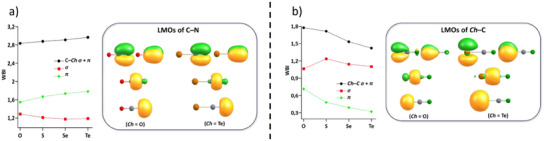
Images of localized molecular valence orbitals (LMOs) of [*Ch*CN]^−^ for the C─N bond a) and the *Ch*–C bond b). For both bonds, images are shown for *Ch* = O and *Ch* = Te, contours are drawn at +/‐ 0.07 a.u. The diagrams left of the LMO images show WBI, also separated into *σ* and *π* contributions, for *Ch* = O, S, Se, Te.

For a deeper insight into chemical bonding, quantum chemical calculations were carried out;^[^ ^18^, ^19^, ^20^, ^21^ ^]^ for details, see ESI. For [*Ch*CN]^−^ (*Ch* = O, S, Se, Te), the *p_z_* and *s* atomic valence orbitals form four occupied *σ*–type molecular orbitals (MOs, for images see Figure S21), and the *p_x_* and *p_y_* orbitals two pairs of *π*–type orbitals. For the simplest possible discussion of the bond situations, *σ*‐ and *π*–type MOs were separately subjected to a localization procedure,^[^ ^22^ ^]^ and the same separation was done in the calculation of Wiberg bond indices (WBI);^[^ ^23^ ^]^ as *σ*‐ and *π*–type orbitals transform according to different irreducible representations, the corresponding WBI add to the total WBI. In Figure 3, the localized MOs (LMOs) are shown for *Ch* = O, Te, and the WBI in total as well as separated into *σ*‐ and *π*–contributions for the C─N and the C─*Ch* bonds. Regardless of *Ch*, the C─N bond shows a WBI close to 3 (Figure 3a), and the LMOs clearly reflect the situation of a triple bond, which can be separated into two bonding *π* LMOs, at the top, and a bonding *σ* LMO, 2nd from top. Additionally, a lone pair at the N atom is found (Figure 3a, bottom). For the C─*Ch* bond, on the other hand (Figure 3b), the WBI is 1.8 for *Ch *= O and decreases to ∼1.4 for *Ch *= Te, mainly due to the WBI of the *π*–system, which decreases from 0.7 to 0.3, as the overlap of C and the chalcogen atom decreases from O to Te. Note, that even for Te the *π–*contribution is nonzero, in accordance with our discussed C─Te bond length (2.051(4)Å), which is slightly shorter than a C─Te single bond (2.11 Å).^[^ ^13^ ^]^ Further note that the total WBI values for the C─S and C─O bonds are similar despite decreasing *π* overlap. This obviously results from stronger *σ*‐bonding between C and O (compared to S, Se, Te), visible by a slight shift of the C─*Ch σ*‐bonding LMO towards C (2nd row of LMOs in Figure 3b). This shift is likely because S, Se, and Te are less electronegative than O.

### NMR Studies

Quaternary carbon atoms usually show weak signals in ^13^C‐NMR spectroscopy, and in our first experiments, we were unable to reliably locate the resonance in [TeCN]^−^. ^14^N‐ and ^15^N‐NMR experiments also proved to be difficult due to intense line broadening of the former and low receptivity of the latter, both additionally hampered by the relatively low solubility of [K@crypt‐222][TeCN]. To allow for a comprehensive NMR spectroscopic investigation, we synthesized [K@crypt‐222][Te^13^C^15^N] from K[^13^C^15^N], tellurium, and crypt‐222. The reaction was performed in acetone‐*d*_6_ at 55°C for 3 h, and the resulting yellowish suspension was filtered into an NMR tube (see Supporting Information for details).

**Figure 4:**
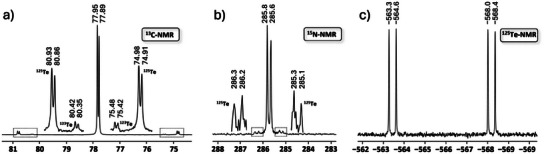
Chemical shifts of [Te^13^C^15^N]^−^ in the ^13^C a), ^15^N b), and ^125^Te c) NMR spectra in ppm. ^13^C Shifts are referenced to (CH_3_)_4_Si, ^15^N are referenced to NH_3_ and ^125^Te to Me_2_Te. ^123^Te and ^125^Te Satellites are shown enlarged. Couplings and their respective values within [Te^13^C^15^N]^−^ are: ^1^ *J*_13C‐125Te_ = 748 Hz, ^1^ *J*_13C‐123Te_ = 621 Hz, ^2^ *J*_15N‐125Te_ = 55 Hz, and ^1^ *J*_13C‐15N_ = 8 Hz.

^1^H‐, ^13^C‐, ^15^N‐, and ^125^Te‐NMR data were collected, and we were able to extract all relevant coupling constants (see Supporting Information Figures S1–S8 for the full spectra). Additionally, chemical shifts and coupling constants were simulated and allowed for the unambiguous determination of the reported values (Figures S9–S11). The ^13^C‐NMR spectrum (Figure 4a) shows a doublet at 77.8 ppm (^1^ *J*_13C‐15N_ = 8 Hz), with ^125^Te (^1^ *J*_13C‐125Te_ = 748 Hz, 7% natural abundance) and ^123^Te (^1^ *J*_13C‐123Te_ = 621 Hz, 1% natural abundance) satellites. In comparison to its lighter chalcogen homologues, we observe significant high field shift (Table 1). The trend from *Ch* = O to *Ch* = Te is not monotonic; the ^13^C shift is highest for [SCN]^−^. The reason for this lies in the electronic structure and the individual contributions to the chemical shift. Details are given below. ^14^N and ^15^N chemical shifts are interchangeable as the primary isotope effect is negligible.^[^ ^24^ ^]^ In the ^15^N NMR spectrum (see Figure 4b), the signal for [TeCN]^−^ is found at 285.7 ppm (referenced to NH_3_) as a doublet (^1^ *J*_13C‐15N_ = 8 Hz). The main signal is flanked by ^125^Te satellites (^2^ *J*_15N‐125Te_ = 55 Hz). In contrast to the ^13^C spectrum, the ^123^Te satellites were too weak to be observed. In the series [*Ch*CN]^−^ with *Ch* = O─Te, the ^15^N chemical shift decreases monotonically (see Table 1). A detailed discussion can be found in the chapter on the DFT calculation of the chemical shifts, see below.

**Table 1:** ^13^C‐ and ^14^N/^15^N‐NMR shifts of [K@crypt‐222][TeCN] and its lighter homologues.

*δ* / ppm	^13^C‐NMR^a)^	^14^N/^15^N‐NMR^b)^	Ref
[OCN]^−^ ^c)^	129.7	76.2	[25]
[SCN]^−^ ^c)^	134.0	206.2	[25]
[SeCN]^−^ ^c)^	120.7	240.2	[25]
[TeCN]^−^	77.8	285.7	this work

^a)^ ^13^C shifts are referenced to tetramethylsilane.

^b)^ ^14^N/^15^N shifts are referenced to liquid NH_3_.

^c)^ Aqueous solution of K[ChCN] at 35 °C.

^125^Te‐NMR spectroscopy (Figure 4c) reveals a doublet of doublets at −566 ppm. Splitting is due to coupling to ^13^C (^1^ *J*_13C‐125Te_ = 748 Hz) and ^15^N (^2^ *J*_15N‐125Te_ = 55 Hz). As the sample was fully ^13^C and ^15^N labeled, all signals show the same intensity. In ^125^Te‐NMR spectroscopy, dimethyl telluride serves as a standard. Chemical shifts of ^125^Te NMR range from approx. −2700 to 1500 ppm, where tellurium compounds with electron‐withdrawing groups have downfield shifts, e.g., Te(CF_3_)_2_ at 1368 ppm. In contrast, electron‐rich tellurium compounds exhibit highfield shifts; Te(Me_3_Sn)_2_, for example, shows a shift of −1214 ppm. Therefore, with a shift of −566 ppm, [TeCN]^−^ can be classified as a moderately shielded tellurium species.

The value for the coupling constant fundamentally depends on the hybridization of the attached carbon atom. The ^1^ *J*_13C‐125Te_ coupling to the *sp*‐hybridized C atom in (RC≡C)_2_Te is 556 Hz, to the *sp* ^2^‐hybridized C atom in (CH_2_═CH)_2_Te is 283.5 Hz, and for the *sp* ^3^‐hybridized C atom in Me_2_Te is 158 Hz. This trend seems to be universal, as has been shown in the literature.^[^ ^26^ ^]^ With a coupling constant of 748 Hz of ^1^ *J*_13C‐125Te_ for the [TeCN]^−^ anion we report the highest ^1^ *J*_13C‐125Te_ coupling constant thus far. As there are no ^2^ *J*_15N‐125Te_ coupling constants in the literature, comparison is not feasible. Hence, with ^2^ *J*_15N‐125Te_ = 55 Hz for [TeCN]^−^ we report the first coupling constant of its kind.

**Figure 5:**
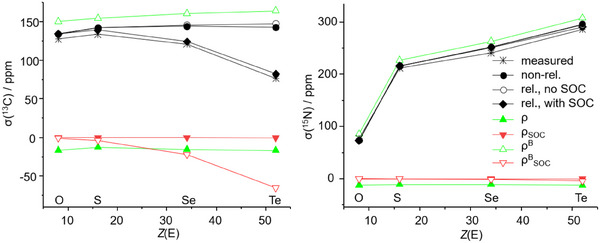
Calculated and measured chemical shifts for ^13^C (w.r.t. TMS, left) and ^15^N (w.r.t. NH_3_, right) in [*Ch*CN]^−^, *Ch* = O, S, Se, Te. Asterisks represent measurements, filled circles non‐relativistic calculations, colorless cycles scalar relativistic calculations (i.e., neglecting SOC), rhombi relativistic calculations with SOC. The latter can be decomposed to their contributions from the unperturbed density (filled triangles) and from the magnetic response density (open triangles), which both can be further decomposed into spin‐orbit‐free parts (green) and spin‐orbit parts (red).

NMR shifts and coupling constants were calculated employing the X2C method (finite‐nucleus approach, screened‐nucleus spin‐orbit approach (SNSO), diagonal local approximation (DLU)^[^ ^20^ ^]^ without^[^ ^27^, ^28^ ^]^ and with^[^ ^29^, ^30^ ^]^ SOC, using x2c‐TZVPall‐2c bases, the density functional PBE0, and the conductor‐like screening model COSMO^[^ ^31^ ^]^ with default parameters for charge compensation. For comparison, also non‐relativistic calculations^[^ ^32^ ^]^ were done with the same functional and basis set. The chemical shifts for ^13^C (w.r.t. TMS) and ^15^N (w.r.t. NH_3_) are shown in Figure 5. For both cases, the shifts calculated, including SOC (black rhombi), very well reproduce the measured data (asterisks). We note in passing that for the reference N compound, NH_3_, the data were calculated for the trimer and averaged to better account for the intermolecular N‐H interactions. For both elements the shifts significantly depend on the type of the chalcogen, but this is for different reasons. For C, agreement is achieved only, if SOC is considered, indicating a so‐called SO‐HALA effect (“SOC induced heavy‐atom effect on the light‐atom shielding”), see for instance ref. [33]. In detail, scalar relativistic calculations (i.e., neglecting SOC, colorless circles) and non‐relativistic treatments (black circles) are very similar to each other but fail even qualitatively for [SeCN]^−^ and are even worse for [TeCN]^−^. The role of SOC becomes even more evident when breaking down the entire shift into its contributions stemming from the non‐perturbed density and the magnetically perturbed density, and each of those into their scalar and spin‐orbit parts (Figure 5). Obviously, it is the spin‐orbit part of the magnetic response density that mainly determines the course of the chemical shift from O to Te by its decrease, superimposed by the slight increase of the scalar part, leading to a maximum for S. Contributions of the unperturbed density are almost constant and, moreover, small. Matters are different for N. Here, non‐relativistic, and relativistic calculations with or without SOC yield almost the same results. The effect of the heavy atom on the shift of the light atom for N is not due to relativity or SOC, but rather due to slight changes in the electronic situation at N (including the C─N bond, see Figure 3). Those cause the changes in the scalar part of the magnetic response that by far dominate the chemical shift (Figure 5). For completeness, we mention the reasonable agreement of the calculated shifts for Se/Te (w.r.t. SeMe_2_/TeMe_2_) amounting to −433/─827 ppm with SOC or −425/−750 ppm without SOC (expt.: −315/−566 pm). The contribution of SOC thus increases from ∼8 ppm or ∼2% of the shift for Se to ∼80 ppm or ∼10% of the shift for Te.

A very reasonable agreement is also found for coupling constants (Table S3). Numbers with and without SOC typically agree within a few Hz, thus the influence of SOC is small; only for the C─Te coupling a reduction by ∼10% due to SOC is found. The coupling constants are dominated by the Fermi‐contact (FC) term, which mainly depends on the spin‐density at the position of the nuclei, as evident from the similarity of numbers obtained with all terms (FC, spin‐dipole and paramagnetic spin‐orbit term) being included and numbers only including the FC term.

### NIPES

**Figure 6:**
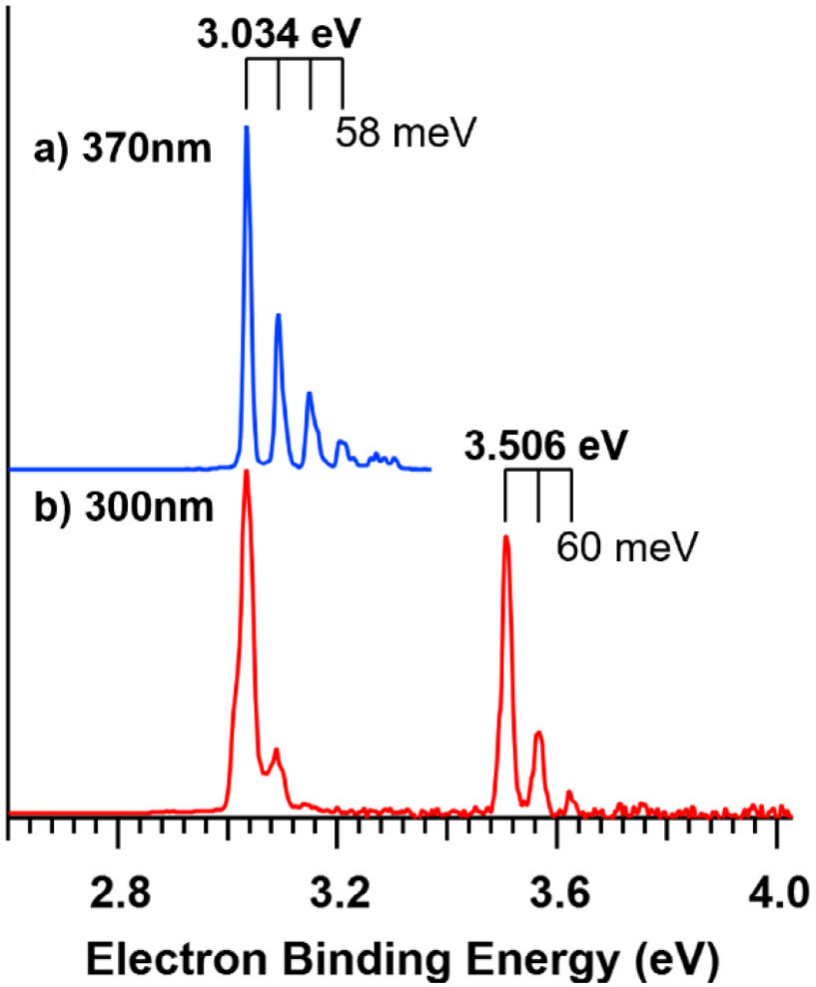
T = 20 K NIPE spectra of the [TeCN]^–^ anion recorded with 370 nm (3.351 eV, a) and 300 nm (4.133 eV, b) photodetachment wavelengths.

NIPES measurements were performed and combined with high‐level quantum‐chemical calculations to give insight into the intrinsic molecular properties of [TeCN]^−^ and its neutral radical molecule [TeCN]^•^. Figure 6 exhibits T = 20 K NIPE spectrum of the [TeCN]^−^ anion recorded at 300 nm (4.133 eV), which is composed of two dominant sharp peaks at 3.034 and 3.506 eV, each of which is followed by a few weaker bands with nearly identical intervals. These two dominant peaks are unambiguously assigned to 0–0 transitions from the anion ground state to ^2^Π_3/2_ and ^2^Π_1/2_ SOC states of its neutral radical, respectively. Due to the clean spectral profile and high resolution that affords the identification of 0–0 transition, the EA of the neutral [TeCN]^•^ molecule is precisely determined to be 3.034(5) eV, whereas the spin‐orbit splitting between ^2^Π_3/2_ and ^2^Π_1/2_ states is measured as 472 meV (3807(20) cm^−1^). Meanwhile, the computed EA of 3.084 eV at the CCSD(T)/aug‐cc‐pVTZ(‐PP) level of theory agrees excellently with the measured value of 3.034 eV, while calculated spin‐orbit splitting of 4105 cm^−1^ based on CASPT2/aug‐cc‐pVTZ(‐DK3) also matches well with the observed splitting of 3807 cm^−1^. Comparing EAs of analogues molecules in the [*Ch*CN]^•^ series, a clearly decreasing trend is found for *Ch* = O (3.609 eV),^[^ ^34^ ^]^ S (3.537 eV),^[^ ^34^ ^]^ Se (3.330 eV),^[^ ^35^ ^]^ and Te (3.034 eV) (see Table 2), which is parallel to that in electronegativities of O (3.44), S (2.58), Se (2.55), and Te (2.1), respectively.^[^ ^36^ ^]^ On the other hand, due to largest nuclear charge in Te, the neutral [TeCN]^•^ molecule displays the largest spin‐orbit splitting of 3807 cm^−1^, compared to those of 95.6, 323.4, and 1653 cm^−1^ in [OCN]^•^,^[^ ^34^ ^]^ [SCN]^•^,^[^ ^34^ ^]^ and [SeCN]^•^,^[^ ^35^ ^]^ respectively. When compared to the related [SeCCH]^•^,^[^ ^37^ ^]^ the SOC is similar to that of [SeCN]^•^. Due to the absence of nitrogen, the EA, however, is lower than in any of the [*Ch*CN]^•^ molecules (Table 2).

**Table 2:** EA in eV and SOC in cm^−1^ of [*Ch*CN]^•^ (*Ch* = O, S, Se, Te) and [SeCCH]^•^.

Species	EA / eV	SOC / cm^−1^	Ref
[OCN]^•^	3.609	95.6	[34]
[SCN]^•^	3.537	323.4	[34]
[SeCN]^•^	3.330	1653	[35]
[TeCN]^•^	3.034	3807	this work
[SeCCH]^•^	2.517	1492	[37]

Moreover, the weak bands coupled to each dominant peak are assigned to a vibrational progression, which is determined to be the C─Te stretch with the assistance of CCSD(T) frequency calculations. From the 300 nm spectrum (Figure 6b), a clear progression with an interval of 484(20) cm^−1^ (60 meV) is observed, representing a C─Te stretching frequency of 484 cm^−1^ in the ^2^Π_1/2_ state. Though not as well resolved in the region of 3.0–3.2 eV on this spectrum, an additional spectrum at 370 nm (3.351 eV, Figure 6a) unambiguously resolves this region and exhibits a clear vibrational progression, from which the C─Te stretch in the ^2^Π_3/2_ ground state is precisely measured as 468(20) cm^−1^ (58 meV). These assignments are based upon excellent agreements with computed C─Te vibrational frequency of 472 cm^−1^ at the CCSD(T)/aug‐cc‐pVTZ(‐PP) level of theory. Among the [*Ch*CN]^•^ series, this frequency also exhibits a decreasing trend upon lighter to heavier *Ch* elements, from 1951 cm^−1^ for [OCN]^•^,^[^ ^34^ ^]^ to 735 cm^−1^ for [SCN]^•^,^[^ ^34^ ^]^ to 565 cm^−1^ for [SeCN]^•^,^[^ ^35^ ^]^ and to 468 cm^−1^ for [TeCN]^•^.

### IR/Raman Spectroscopy

Vibrational spectra (IR and Raman) were recorded for [K@crypt‐222][TeCN] (see Supporting Information for experimental details). The IR spectrum is dominated by spectroscopic signatures of the cryptand (Figures S12, S13), whereas the Raman spectrum shows one strong signal attributed to the CN vibration of the [TeCN]^−^ anion, alongside some broad signals between 2800–3000 cm^−1^ that stem from vibrations of the C─H groups of the cryptand (Figures S12, S14, and S15). In principle, [TeCN]^−^ gives three IR and Raman active bands: The asymmetric and symmetric stretching vibrations, and the deformation vibration. In comparison to the C and N atoms, the Te atom is very heavy and consequently, the symmetric stretching vibration is dominated by motions of the C and N atoms, giving characteristic signals at 2073 cm^−1^ in the Raman and at 2074 cm^−1^ in the IR spectrum. Those values are similar to reported bands for [PPN][TeCN], [Ph_4_P][TeCN], and [Ph_4_As][TeCN].^[^ ^38^ ^]^ In contrast, the asymmetric stretching vibration and deformation vibration bands are weak. For the aforementioned compounds, the bands can be found at approximately. 450 and 340 cm^−1^, but are in our case somewhat encumbered by bands of cryptand‐222. One could perhaps locate these bands in the IR spectrum as very low intensity signals at 463 and 450 cm^−1^, see the insert of Figure S12.

## Conclusion

The reaction of KCN with tellurium and crypt‐222 in acetone quantitatively yields [K@crypt‐222][TeCN]. In our work, we investigated its single crystal structure, NMR, and vibrational (Raman and IR) spectroscopic signatures, and described important trends in NMR and bonding situation corroborated by computational studies. Additionally, for the neutral [TeCN]^•^ molecule, we discuss its EA and vibrational signature as measured by negative ion photoelectron spectroscopy.

In [K@crypt‐222][TeCN] the anion is not coordinated to the cation. Bond lengths of the [TeCN]^−^ anion indicate a C≡N triple bond, and the C─Te bond length indicates multiple bond sharing with a bond order between 1 and 2. These findings are in accordance with their calculated WBIs of 2.96 and 1.42, respectively. In the series [*Ch*CN]^−^ (*Ch *= O‐Te), bond order between C and Te decreases monotonically. Differentiation into *σ*‐ and *π*–bonding shows a maximum in *σ*‐bonding for *Ch* = S, whereas the *π*–bonding decreases monotonically.

Synthesis of [K@crypt‐222][Te^13^C^15^N] allowed for the full characterization by ^13^C, ^15^N and ^125^Te NMR spectroscopy and simulation of the spectra for extraction of the coupling constants (^13^C = 77.8 ppm, ^15^N = 285.7 ppm, ^125^Te = −566 ppm, ^1^ *J*_13C‐15N_ = 8 Hz, ^1^ *J*_13C‐125Te_ = 748 Hz, ^2^ *J*_15N‐125Te_ = 55 Hz). NMR studies were supported by quantum mechanical calculations. We find that the influence of the chalcogen atom is remarkable for the ^13^C shifts (SO‐HALA effect). However, this effect is not responsible for the alteration of the ^15^N shifts, since these are also well described without considering relativistic effects and/or SOC. Rather, it is the alteration of the electronic situation of the C─N bond within [*Ch*CN]^−^ (*Ch* = O, S, Se) that causes *Ch*‐dependent differences in the shift‐dominating scalar part of the magnetic response density. NIPES shows the EA of the neutral [TeCN]^•^ molecule to be 3.034 eV, which is smallest in the monotonic series [*Ch*CN]^•^ (*Ch* = O‐Te), which is in accordance with the respective electronegativities of the chalcogen atoms. On the contrary, the SOC in neutral [TeCN]^•^ is largest in that series with a splitting of 3807 cm^−1^. Vibrational progressions from the 0–0 transition allow for the extraction of the C─Te stretching vibration of neutral [TeCN]^•^ which, measured at 370 nm is 468 cm^−1^. Studies utilizing [K@crypt‐222][TeCN] as a synthon to expand the scope of [TeCN]⁻ chemistry are currently in progress.

## Author Contributions

H.G. is responsible for the synthesis and collection of data (X‐ray, IR/Raman). F.W. is responsible for the calculations for chemical bonding analysis, NMR shielding, and NMR coupling tensors. X.X. is responsible for the collection and simulation of NMR data. W.C., X.‐F.G., and X.‐B.W. are responsible for the NIPES studies and associated calculations. F.T. is responsible for conceptualization and project lead. All authors have participated in the writing of the draft, the review processes, and have given their approval to the final version of the manuscript.

## Conflict of Interests

The authors declare no conflict of interest.

## Supporting information



Supporting Information

Supporting Information

## Data Availability

The data that support the findings of this study are available in the supplementary material of this article.

## References

[1] L. Birckenbach , K. Kellermann , Ber. dtsch. Chem. Ges. A/B 1925, 58, 786–794.

[2] G. Becker , W. Schwarz , N. Seidler , M. Westerhausen , Z. Anorg. Allg. Chem. 1992, 612, 72–82.

[3] G. Becker , K. Hübler , Z. Anorg. Allg. Chem. 1994, 620, 405–417.

[4] A. Hinz , J. M. Goicoechea , Angew. Chem., Int. Ed. 2016, 55, 8536–8541. 10.1002/anie.201602310 PMC5074235 27093942

[5] F. Tambornino , A. Hinz , R. Köppe , J. M. Goicoechea , Angew. Chem., Int. Ed. 2018, 57, 8230–8234. 10.1002/anie.201805348 29786936

[6] J. M. Goicoechea , H. Grützmacher , Angew. Chem., Int. Ed. 2018, 57, 16968–16994. 10.1002/anie.201803888 29770548

[7] L. Birckenbach , K. Huttner , Z. Anorg. Allg. Chem. 1930, 190, 1–26.

[8] A. W. Downs , Chem. Commun. (London) 1968, 1290.

[9] A. Martinsen , J. Songstad , R. Larsson , M. Pouchard , P. Hagenmuller , A. F. Andresen , Acta Chem. Scand. 1977, 31a, 645–650.

[10] A. S. Foust , J. Chem. Soc., Chem. Commun. 1979, 414.

[11] N. A. Semenov , A. V. Lonchakov , N. A. Pushkarevsky , E. A. Suturina , V. V. Korolev , E. Lork , V. G. Vasiliev , S. N. Konchenko , J. Beckmann , N. P. Gritsan , A. V. Zibarev , Organometallics 2014, 33, 4302–4314.

[12] H. K. Spencer , M. V. Lakshmikantham , M. P. Cava , J. Am. Chem. Soc. 1977, 99, 1470–1473.

[13] P. Pyykkö , M. Atsumi , Chem. – Eur. J. 2009, 15, 12770–12779. 19856342 10.1002/chem.200901472

[14] M. Bujak , H. Stammler , N. W. Mitzel , Chem. – Eur. J. 2025, 31, e202404648. 39821385 10.1002/chem.202404648

[15] S. V. Rosokha , C. L. Stern , A. Swartz , R. Stewart , Phys. Chem. Chem. Phys. 2014, 16, 12968–12979. 24852189 10.1039/c4cp00976b

[16] H. Bock , S. Holl , Z. Naturforsch. B 2002, 57, 843.

[17] Nuzzo, S. , Twamley, B. , Baker, R. J. , CSD Communication Deposition Number 2289513, 2023, 10.5517/CCDC.CSD.CC2GVF81.

[18] TURBOMOLE, Version 7.9 2024; a development of University of Karlsruhe and Forschungszentrum Karlsruhe GmbH 1989–2007, TURBOMOLE GmbH since 2007, available via https://www.turbomole.org 2024.

[19] J. P. Perdew , M. Ernzerhof , K. Burke , J. Chem. Phys. 1996, 105, 9982–9985.

[20] Y. J. Franzke , N. Middendorf , F. Weigend , J. Chem. Phys. 2018, 148, 104110. 29544265 10.1063/1.5022153

[21] P. Pollak , F. Weigend , J. Chem. Theory Comput. 2017, 13, 3696–3705. 28679044 10.1021/acs.jctc.7b00593

[22] J. Pipek , P. G. Mezey , J. Chem. Phys. 1989, 90, 4916–4926.

[23] K. B. Wiberg , Tetrahedron 1968, 24, 1083–1096.

[24] E. D. Becker , R. B. Bradley , T. Axenrod , J. Magn. Reson. 1971, 4, 136–141.

[25] J. A. Kargol , R. W. Crecely , J. L. Burmeister , Inorg. Chem. 1979, 18, 2532–2535.

[26] R. W. Gedridge , K. T. Higa , R. A. Nissan , Magn. Reson. Chem. 1995, 33, 441–448.

[27] Y. J. Franzke , F. Weigend , J. Chem. Theory Comput. 2019, 15, 1028–1043. 30620588 10.1021/acs.jctc.8b01084

[28] Y. J. Franzke , J. Chem. Theory Comput. 2023, 19, 2010–2028. 36939092 10.1021/acs.jctc.2c01248

[29] Y. J. Franzke , C. Holzer , J. Chem. Phys. 2023, 159, 184102. 37937936 10.1063/5.0171509

[30] Y. J. Franzke , F. Mack , F. Weigend , J. Chem. Theory Comput. 2021, 17, 3974–3994. 34151571 10.1021/acs.jctc.1c00167

[31] A. Pausch , J. Chem. Theory Comput. 2024, 20, 3169–3183. 38557008 10.1021/acs.jctc.4c00052

[32] K. Reiter , F. Mack , F. Weigend , J. Chem. Theory Comput. 2018, 14, 191–197. 29232503 10.1021/acs.jctc.7b01115

[33] J. Vícha , J. Novotný , S. Komorovsky , M. Straka , M. Kaupp , R. Marek , Chem. Rev. 2020, 120, 7065–7103. 32574047 10.1021/acs.chemrev.9b00785

[34] S. E. Bradforth , E. H. Kim , D. W. Arnold , D. M. Neumark , J. Chem. Phys. 1993, 98, 800–810.

[35] Q. Yuan , F. Tambornino , A. Hinz , W. T. Borden , J. M. Goicoechea , B. Chen , X.‐B. Wang , Angew. Chem., Int. Ed. 2019, 58, 15062–15068. 10.1002/anie.201906904 31393658

[36] D. R. Lide , G. Baysinger , S. Chemistry , H. V. Kehiaian , L. I. Berger , K. Kuchitsu , R. N. Goldberg , D. L. Roth , M. Library , W. M. Haynes , D. Zwillinger , CRC Handbook of Chemistry and Physics, CRC Press/Taylor And Francis, Boca Raton, FL, 2008.

[37] Q. Yuan , W. Cao , M. Hetzert , U. Ruschewitz , X.‐B. Wang , J. Phys. Chem. A 2020, 124, 3214–3219. 32250629 10.1021/acs.jpca.0c01936

[38] P. Klæboe , C. J. Nielsen , J. Songstad , M. Pouchard , P. Hagenmuller , A. F. Andresen , Acta Chem. Scand. 1977, 31a, 884–886.

