# Supplementary material for: A Thorough Characterization of the Tellurocyanate Anion
Source: Angew Chem Int Ed Engl. 2025 Jun 3;64(31):e202507543. doi: 10.1002/anie.202507543 (PMC12304862; doi:10.1002/anie.202507543)
Supplement: Supplementary file 1 — Supporting Information [file ANIE-64-e202507543-s002.pdf]

Electronic Supporting Information (ESI)

A Thorough Characterization of the Tellurocyanate Anion

Hennes Günther[a], Florian Weigend[b], Xiulan Xie[a], Wenjin Cao[c], Xiao-Fei Gao[c], Xue-Bin Wang[c] and Frank Tambornino[a]*

[a] Department of Chemistry
Philipps University of Marburg
Hans-Meerwein-Str. 4, D-35043 Marburg, Germany
E-mail: frank.tambornino@chemie.uni-marburg.de

[b] Institute for Quantum Materials and Technology
Karlsruhe Institute of Technology
Hermann-von-Helmholtz-Platz 1, D-76344 Eggenstein-Leopoldshafen, Germany

[c] Physical Sciences Division
Pacific Northwest National Laboratory
Richland, WA 99352, USA

Contents

1. Experimental section 3
1.1 General remarks 3
1.2 Additional characterization techniques: 3
1.3 Synthesis of [K@crypt-222][TeCN] 4
1.4 Synthesis of [K@crypt-222][Te^{13}C^{15}N] 4
1.5 Details on NMR spectroscopy on [K@crypt-222][Te^{13}C^{15}N] 4
1.6 Collected NMR spectra of [K@crypt-222][Te^{13}C^{15}N] 6
1.7 Simulated NMR spectra of [K@crypt-222][Te^{13}C^{15}N] 10
1.8 IR spectra of [K@crypt-222][TeCN] 12
1.9 Raman spectra of [K@crypt-222][TeCN] 13
2. Crystallographic data 14
2.1 Single crystal X-ray data collection and refinement parameters 15
2.2 Additional Pictures of [K@crypt-222][TeCN] 16
3. Comparison of [TeCN]$^{–}$ with comparable [*Ch*CN]$^{–}$ in literature 18
4. Details on quantum chemical calculations 19
5. Negative ion photoelectron spectroscopy (NIPES) of [K@crypt-222][TeCN] 22
5.1 Experimental methods 22
5.2 Theoretical methods 22
6. References 24

1. Experimental section

1.1 General remarks

General synthetic methods. All reactions and manipulations were performed under an inert atmosphere of argon using standard Schlenk-line or glovebox techniques (MBraun UNIlab glovebox, maintained at < 0.1 ppm H_2O and < 0.1 ppm O_2).

Solvents were dried according to literature.[1] $C(O)(CD_3)_2$ was degassed and stored over molecular sieve (4 Å) prior to use.

$K[^{13}C^{15}N]$ (99 atom % ^{13}C, 98 atom % ^{15}N) was purchased from Merck, crypt-222 (98%) from Merck and Te (99.8%) from Acros Organics.

1.2 Additional characterization techniques:

Elemental analyses were performed by the in-house service personnel. CHN(S) analyses were performed on a *CHNS(S)-Analysator vario MICRO CUBE* (Elementar).

IR spectra were recorded on a Bruker Alpha FT-IR spectrometer equipped with a diamond ATR unit mounted. The spectrometer was housed in a glovebox (MBraun UNIlab glovebox, maintained at < 0.1 ppm H_2O and < 0.1 ppm O_2).

Raman spectra were measured with a *Monovista CRS+ confocal Raman microscope* (Spectroscopy & Imaging GmbH) equipped with a 633 nm solid-state laser and a 300 grooves/mm (low-resolution mode, FWHM: <4.62 cm−1) grating. For recording samples were flame sealed in borosilicate glass ampoules.

1.3 Synthesis of [K@crypt-222][TeCN]

Potassium cyanide (45.9 mg, 705.3 µmol, 1.00 eq.), tellurium (90.0 mg, 705.33 µmol, 1.00 eq.) and crypt-222 (287.0 mg, 762.3 µmol, 1.08 eq.) were suspended in acetone (5 mL). The dark grey suspension was heated to 60 °C for 2.5 h and filtered through a filter cannula (pore size: 0.2 µm, PVDF filter media). The resulting yellowish solution was dried *in vacuo* which afforded the product as light grey solid contaminated with a slight excess of crypt-222. The amount of isolated product mixture was 420 mg, corresponding to a theoretical yield of ca. 105%; for 100% the theoretical yield is 401.5 mg. We assume that the reaction between cyanide and tellurium was quantitative, and the surplus is due to the excess of crypt-222 used. The product was stored at −30 °C under exclusion of light.

Element analysis (calc./found.) C: 40.09 / 40.96, N: 7.38 / 7.41, H: 6.38 / 6.87.

1.4 Synthesis of [K@crypt-222][Te^{13}C^{15}N]

^{13}C and ^{15}N labelled potassium cyanide (10.00 mg, 149.03 µmol, 1.00 eq.), tellurium (19.02 mg, 149.03 µmol, 1.00 eq.) and crypt-222 (56.11 mg, 149.03 µmol, 1.00 eq.) were suspended in acetone-d_6 (0.5 mL). The dark grey suspension was heated to 55 °C for 3 h. The resulting yellowish suspension with little grey precipitate was filtered through a filter cannula (pore size: 0.2 µm, PVDF filter media) into an NMR tube and was sealed with a septum.

1.5 Details on NMR spectroscopy on [K@crypt-222][Te^{13}C^{15}N]

Spectra were recorded on a Bruker AVIII 500 MHz spectrometer installed with a 5 mm Cryoprobe Prodigy BBO. Chemical shift of ^{13}C was referenced to the solvent signal, while that of ^{125}Te to neat Me_2Te and ^{15}N to liquid NH_3 externally. In order to gain enough quality for the observation of the $^{123/125}$Te satellites, the ^{13}C and ^{15}N spectra were acquired with 512 transients. All spectra were acquired with 30 pulses, while relaxation delays for ^{125}Te and ^{15}N were 3 s

and 16 s, respectively. Spectra were processed with Bruker program Topspin 4.4.0, while spectra simulation was done with Daisy, a fitting module within Topspin.

^{1}H NMR (300 MHz, acetone-d_6, r.t.) δ / ppm = 3.63 (s, 12H, 6xC*H*2), 3.60 (t, 12H, 6xC*H*2), 2.60 (t, 12H, 6xC*H*2).

^{13}C NMR (125.8 MHz, acetone-d_6, r.t.) δ / ppm = 77.92 (d, $^{1}J_{13C\text{-}15N}$ = 8 Hz, $^{1}J_{13C\text{-}125Te}$ = 748 Hz, $^{1}J_{13C\text{-}123Te}$ = 621 Hz, 1C, $[Te^{13}C^{15}N]^{-}$), 71.0 (s, 4C, 6x*C*H2), 68.2 (s, 4C, 6x*C*H2), 54.5 (s, 4C, 6x*C*H2).

^{15}N NMR (50.7 MHz, acetone-d_6, r.t.) δ / ppm = 285.72 (d, $^{1}J_{15N13C}$ = 8 Hz, $^{2}J_{15N\text{-}125Te}$ = 55 Hz, 1N, $Te^{13}C^{15}N$).

^{125}Te NMR (157.8 MHz, acetone-d_6, r.t.) δ / ppm = −565.8 (dd, $^{1}J_{13C\text{-}125Te}$ = 748 Hz, $^{2}J_{15N\text{-}125Te}$ = 55 Hz, 1Te, $Te^{13}C^{15}N$).

1.6 Collected NMR spectra of [K@crypt-222][$Te^{13}C^{15}N$]

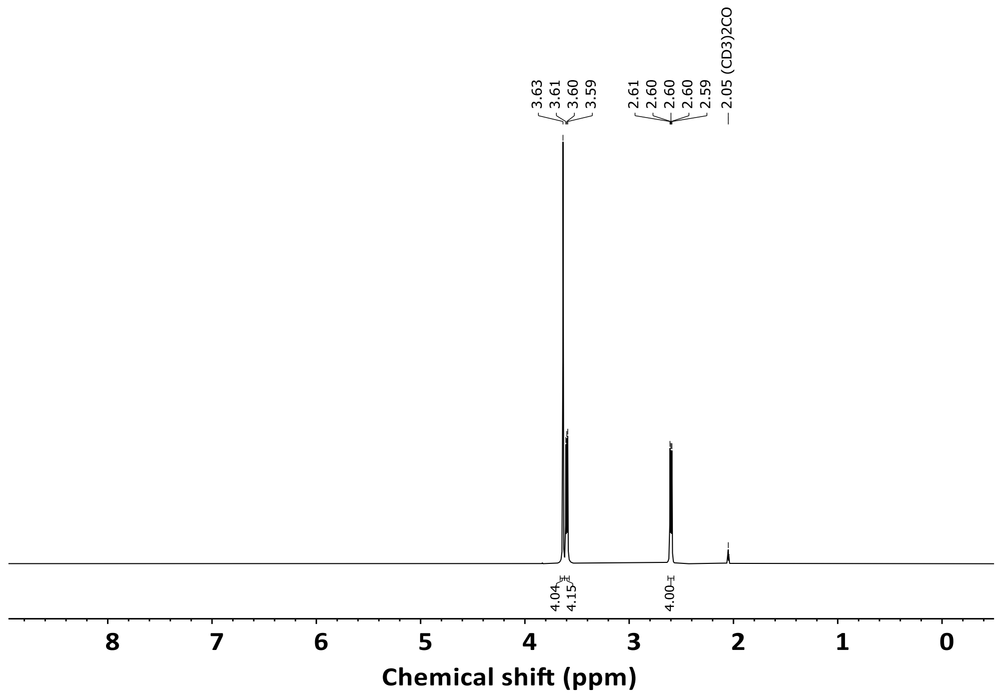

Figure S 1: ^{1}H-NMR spectrum of [K@crypt-222][$Te^{13}C^{15}N$] in acetone-d_6.

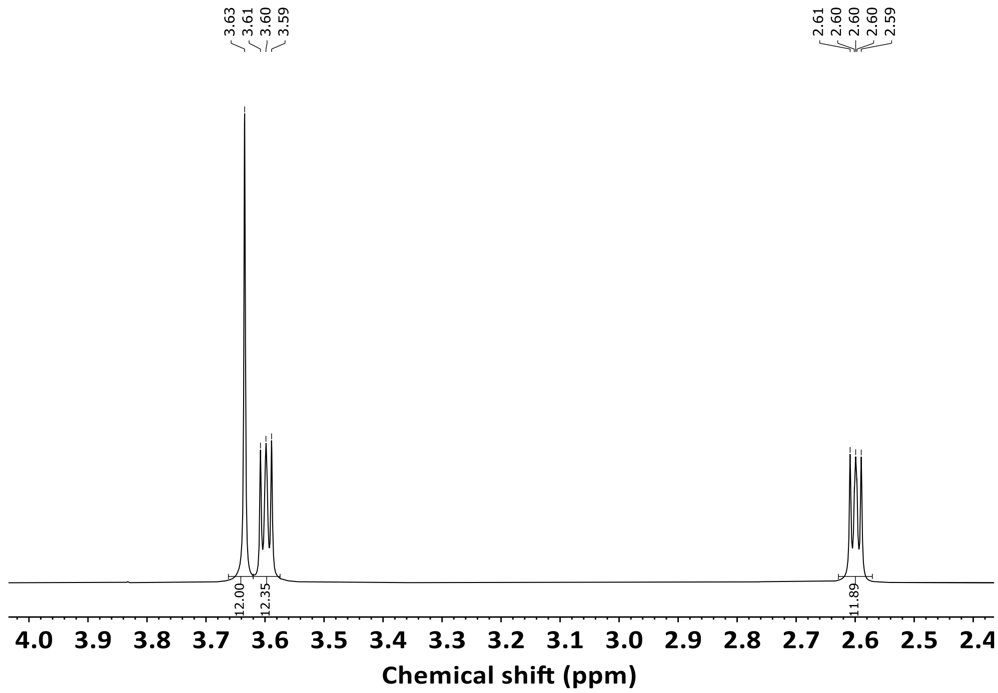

Figure S 2: Enlargement of crypt-222 signals in the ^{1}H-NMR spectrum of [K@crypt-222][$Te^{13}C^{15}N$] in acetone-d_6.

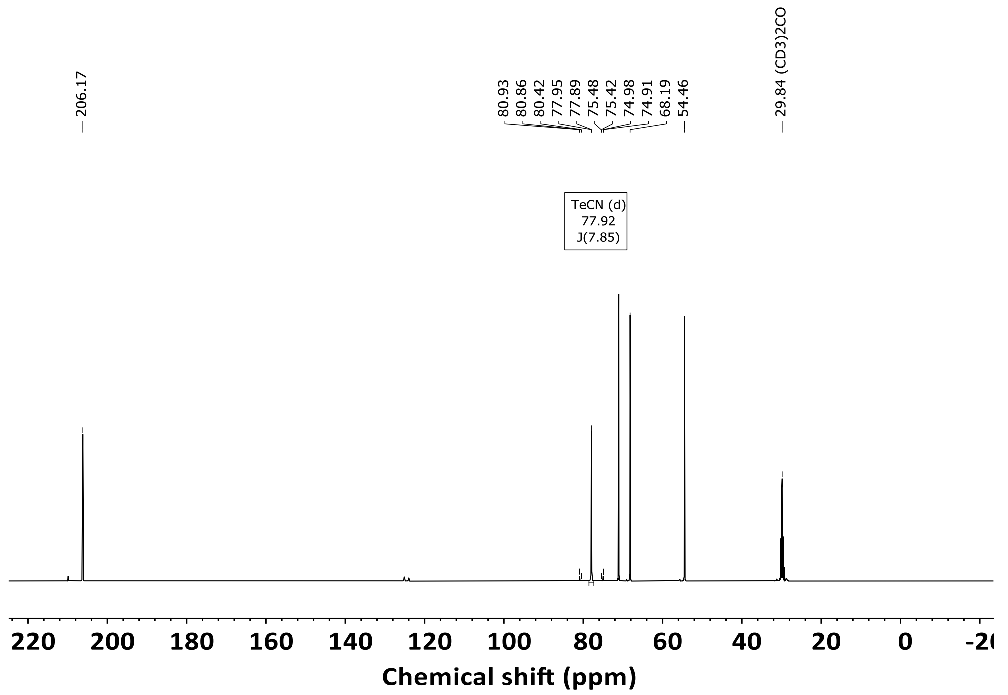

Figure S 3: ^{13}C-NMR spectrum of [K@crypt-222][Te^{13}C^{15}N] in acetone-d_6.

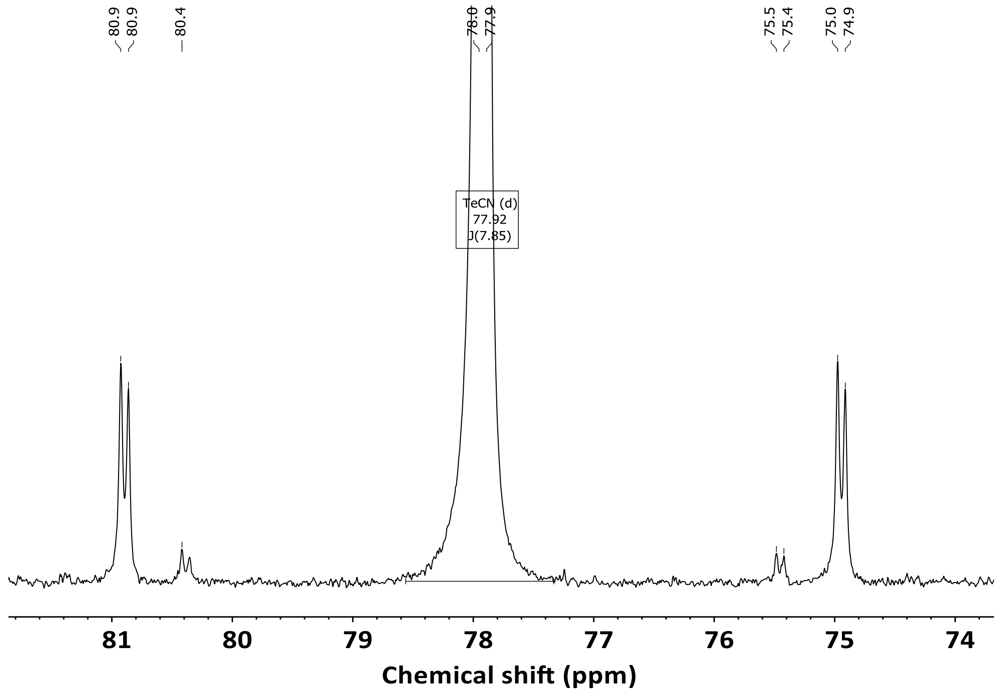

Figure S 4: Enlargement of [Te*C*N]$^{-}$ signal with ^{125}Te, ^{123}Te satellites in the ^{13}C-NMR spectrum of [K@crypt-222][Te^{13}C^{15}N] in acetone-d_6.

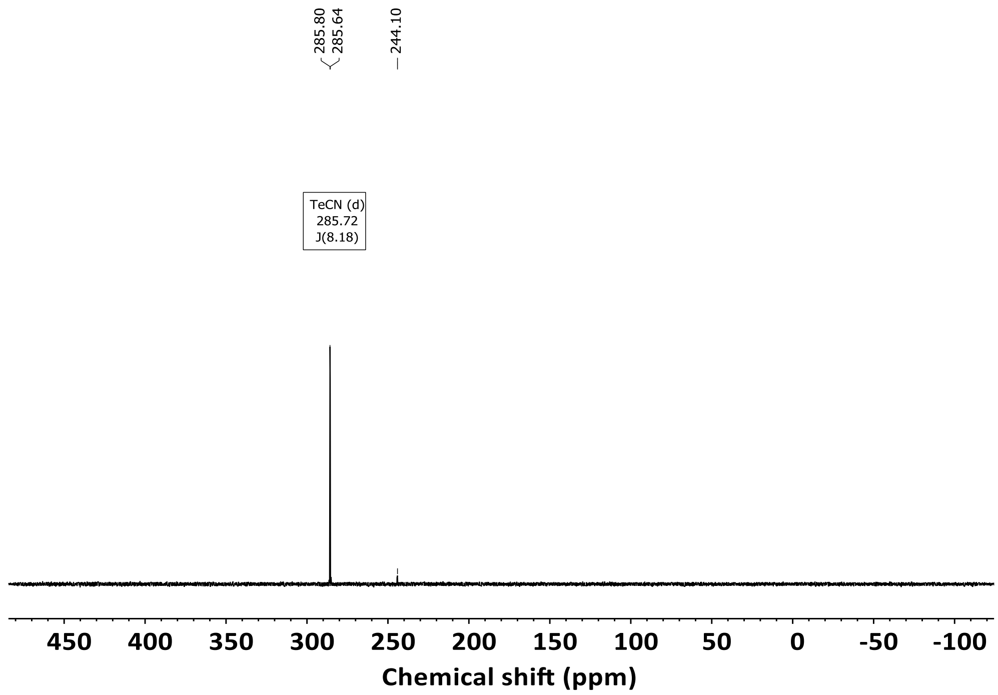

Figure S 5: ^{15}N-NMR spectrum of [K@crypt-222][$Te^{13}C^{15}N$] in acetone-d_6.

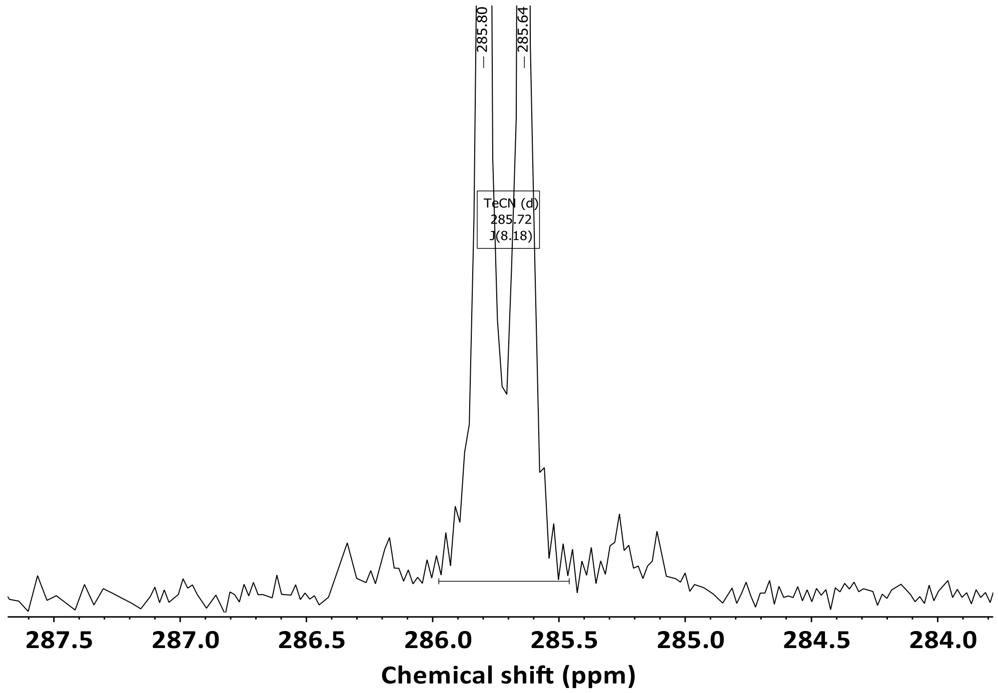

Figure S 6: Enlargement of $[Te^{13}C^{15}N]^-$ signal with ^{125}Te satellites in the ^{15}N-NMR spectrum of [K@crypt-222][$Te^{13}C^{15}N$] in acetone-d_6.

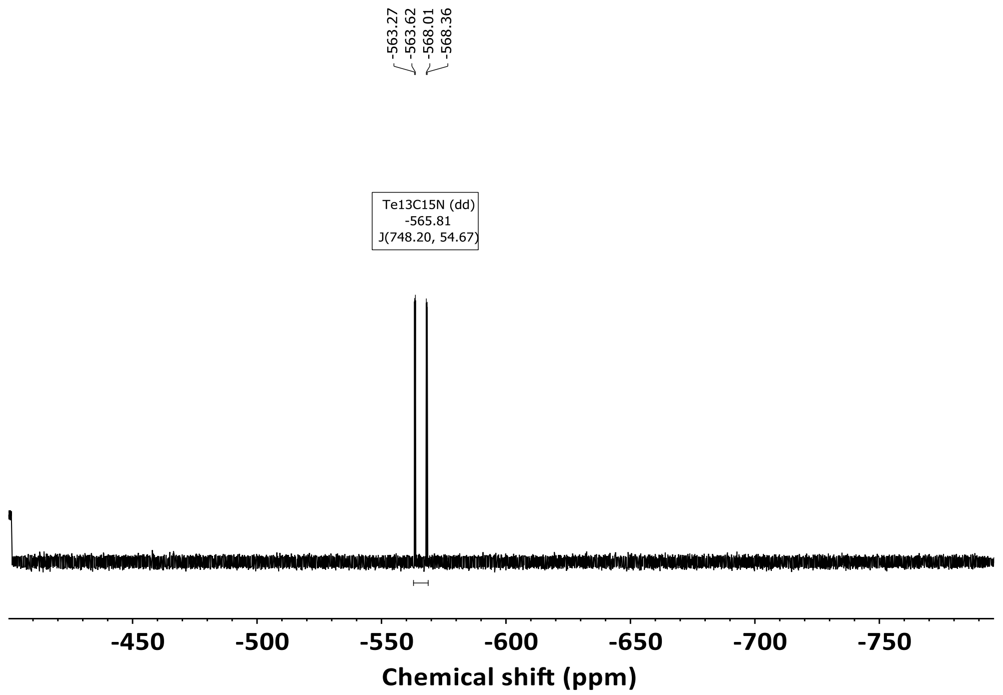

Figure S 7: ^{125}Te-NMR spectrum of [K@crypt2.2.2][Te^{13}C^{15}N] in acetone-d_6.

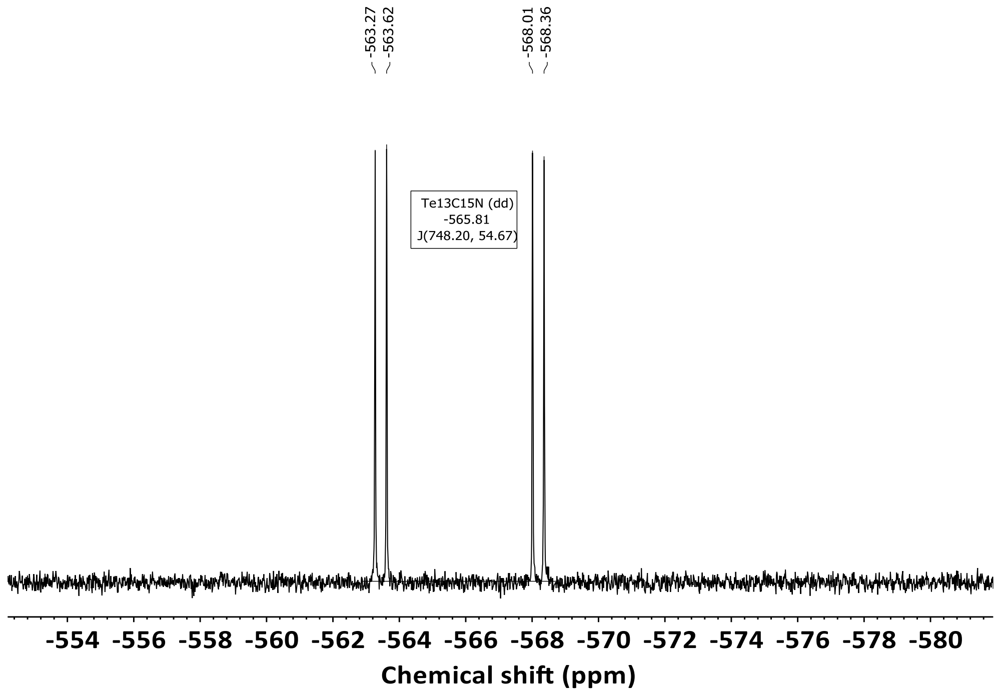

Figure S 8: Enlargement of [*Te*^{13}C^{15}N]$^{-}$ signal ^{125}Te-NMR spectrum of [K@crypt-222][Te^{13}C^{15}N] in acetone-d_6.

1.7 Simulated NMR spectra of [K@crypt-222][Te^{13}C^{15}N]

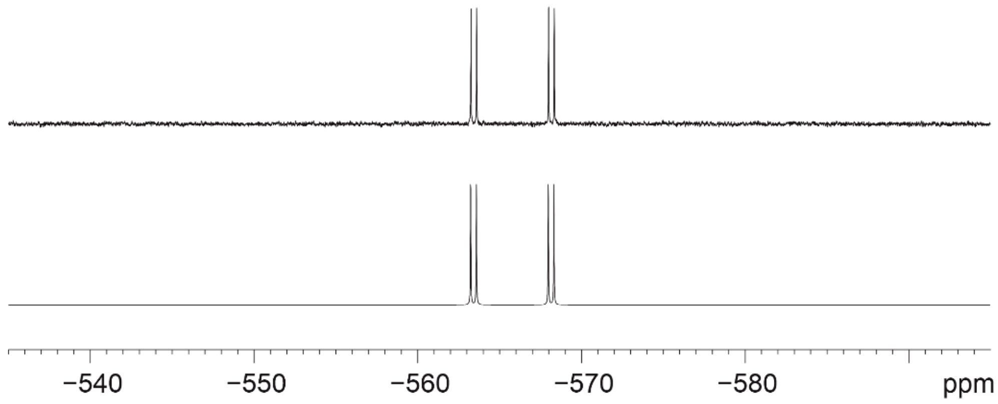

Figure S 9: The ^{125}Te spectra (157.8 MHz, upper trace experimental and lower trace simulated) of [Te^{13}C^{15}N] in acetone-d_6 at 298 K. The observed doubled doublets were caused by coupling with the fully isotope labelled ^{13}C and ^{15}N, respectively.

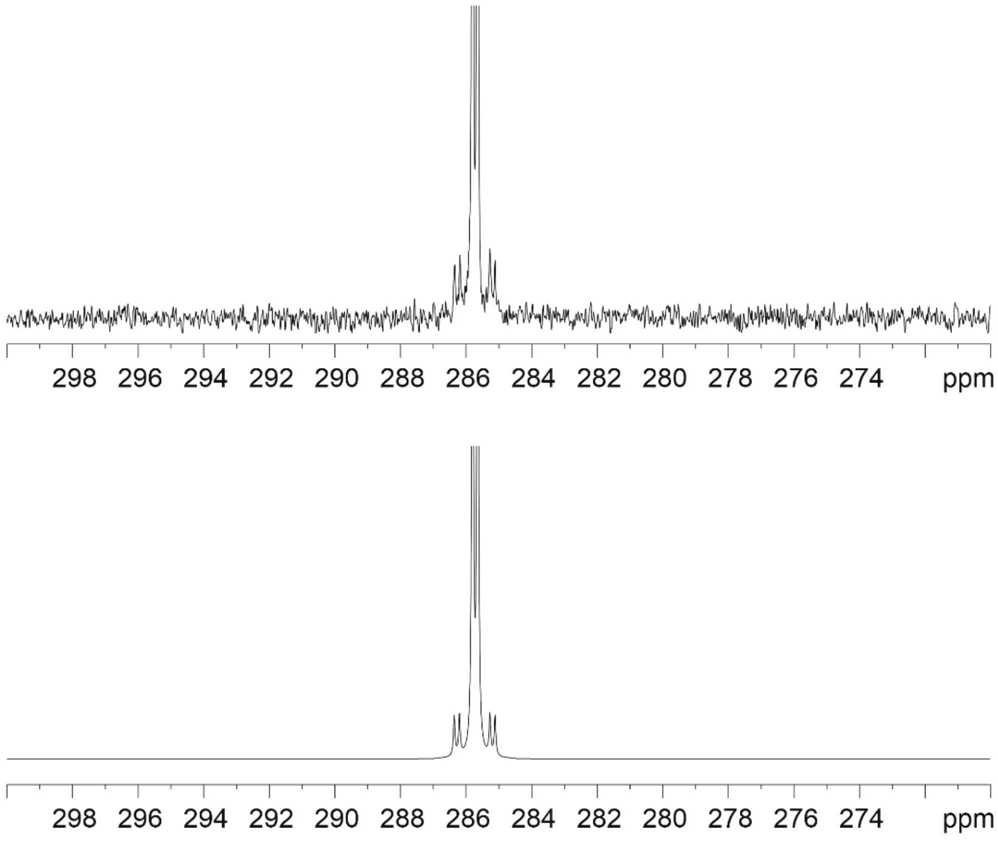

Figure S 10: The ^{15}N spectra (50.7 MHz, upper trace experimental and lower trace simulated) of [Te^{13}C^{15}N] in acetone-d_6 at 298 K. The central doublet was due to coupling with the fully isotope labelled ^{13}C, while the shoulder in doublets were ^{125}Te satellites coupled with isotope ^{13}C.

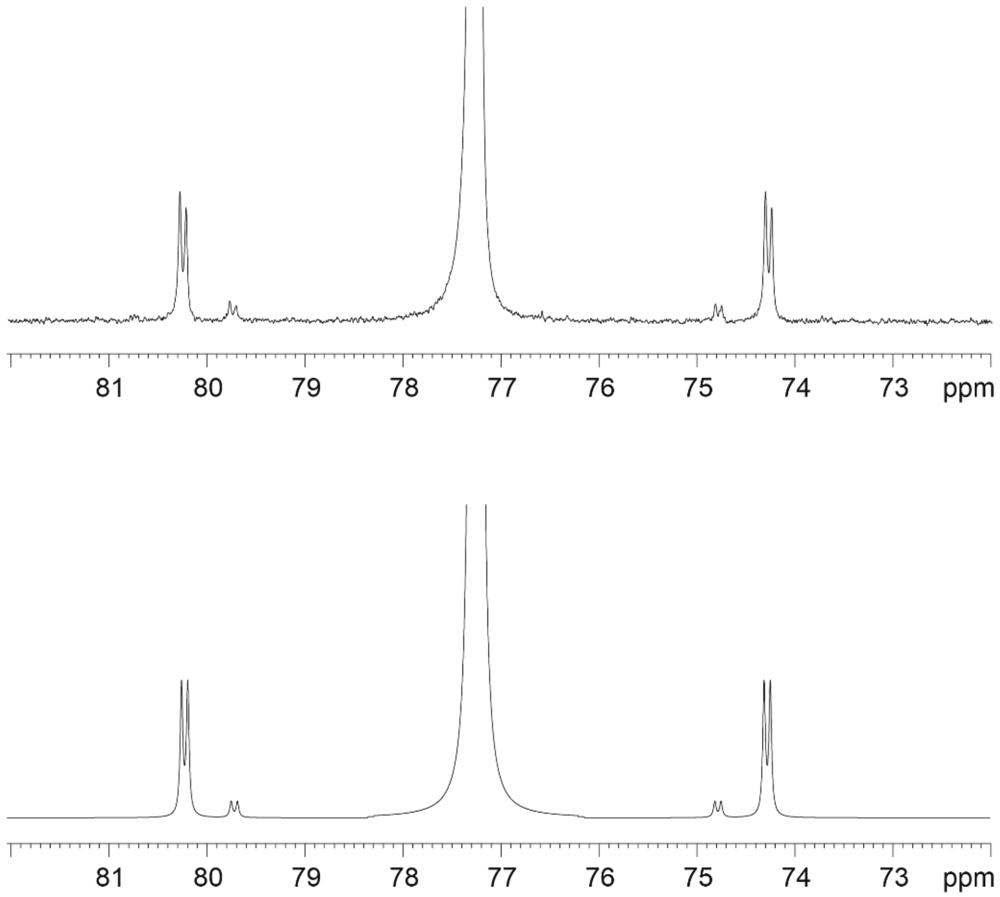

Figure S 11: The ^{13}C spectra (125.8 MHz, upper trace experimental and lower trace simulated) of $[Te^{13}C^{15}N]$ in acetone-d_6 at 298 K. Two sets of satellites due to ^{125}Te and ^{123}Te, respectively were observed. The doublets were caused by coupling with the fully isotope labelled ^{15}N.

1.8 IR spectra of [K@crypt-222][TeCN]

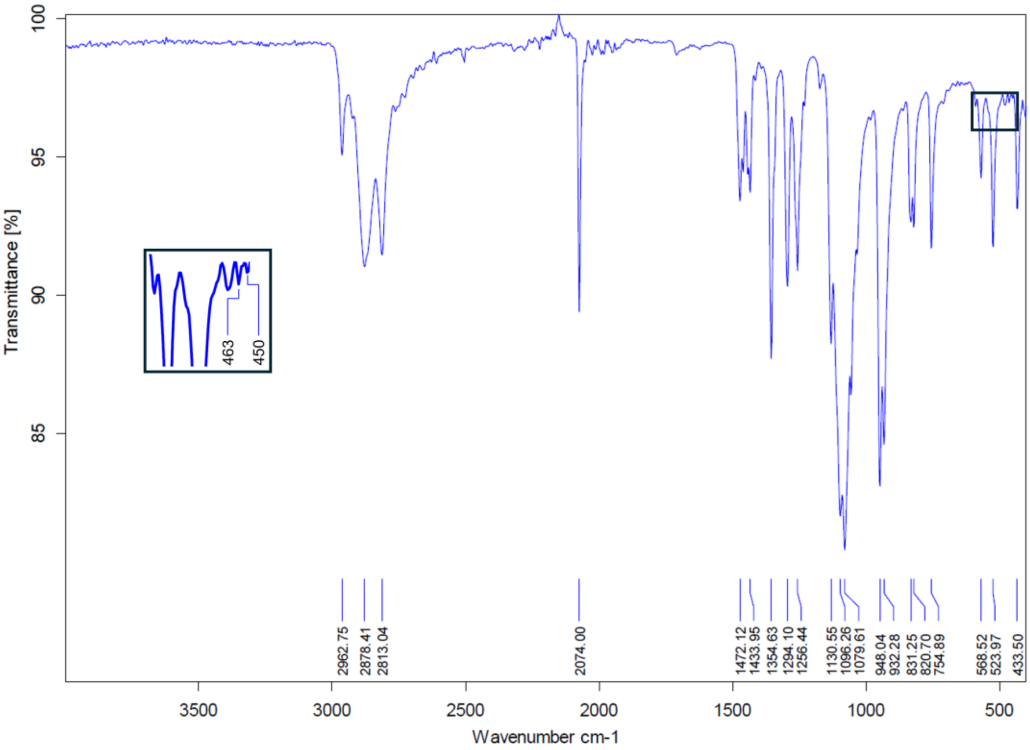

Figure S 12: IR spectrum of [K@crypt-222][TeCN] measured at room temperature with enlargement of signals at 463 cm^{-1} and 450 cm^{-1}.

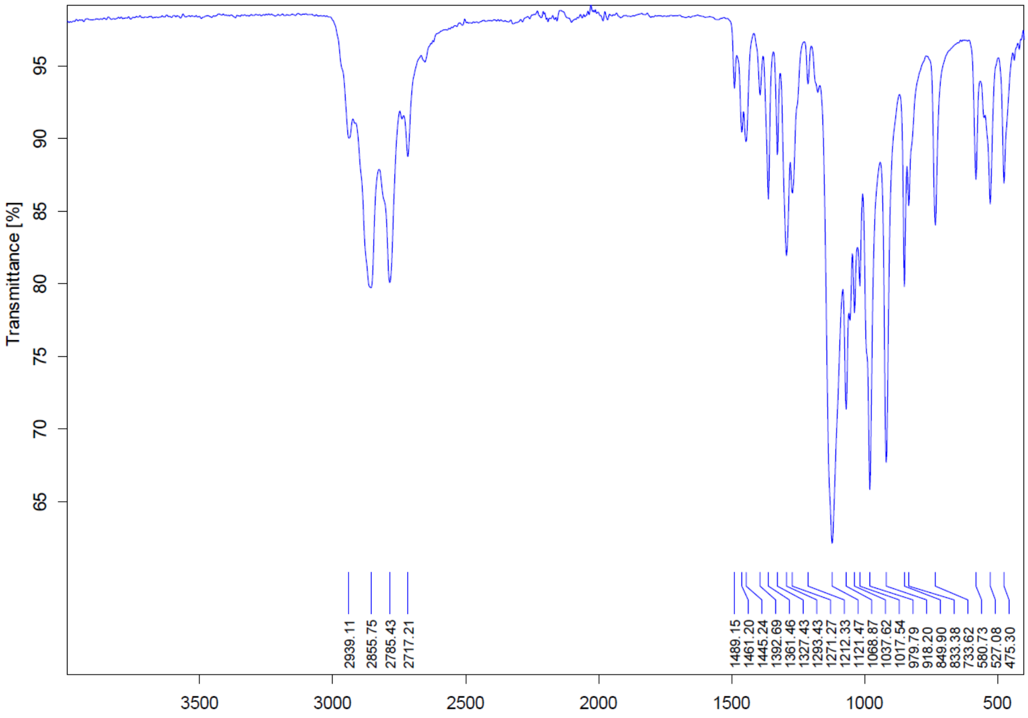

Figure S 13: IR spectrum of crypt-222 measured at room temperature.

1.9 Raman spectra of [K@crypt-222][TeCN]

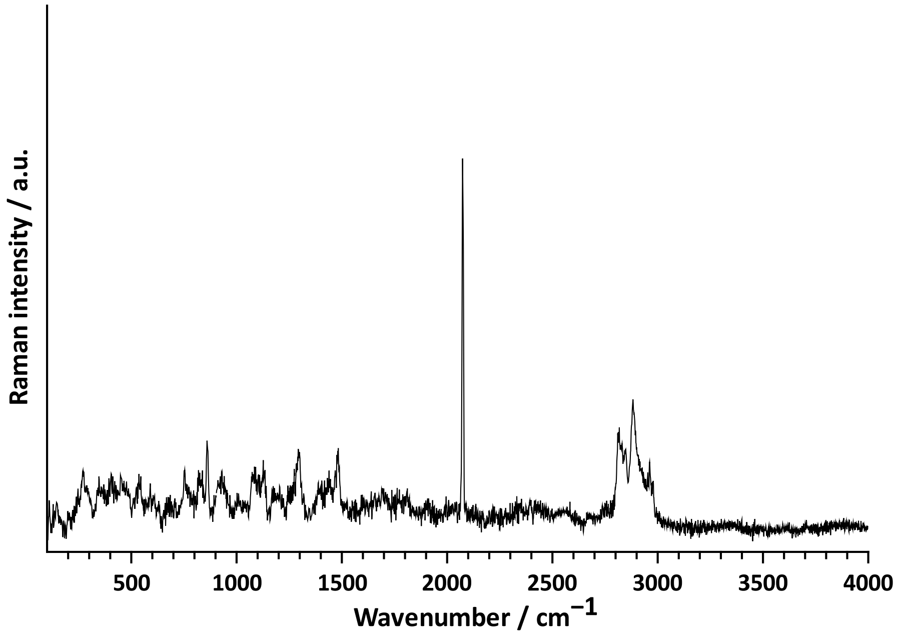

Figure S 14: Raman spectrum of [K@crypt-222][TeCN] measured at room temperature after baseline correction.

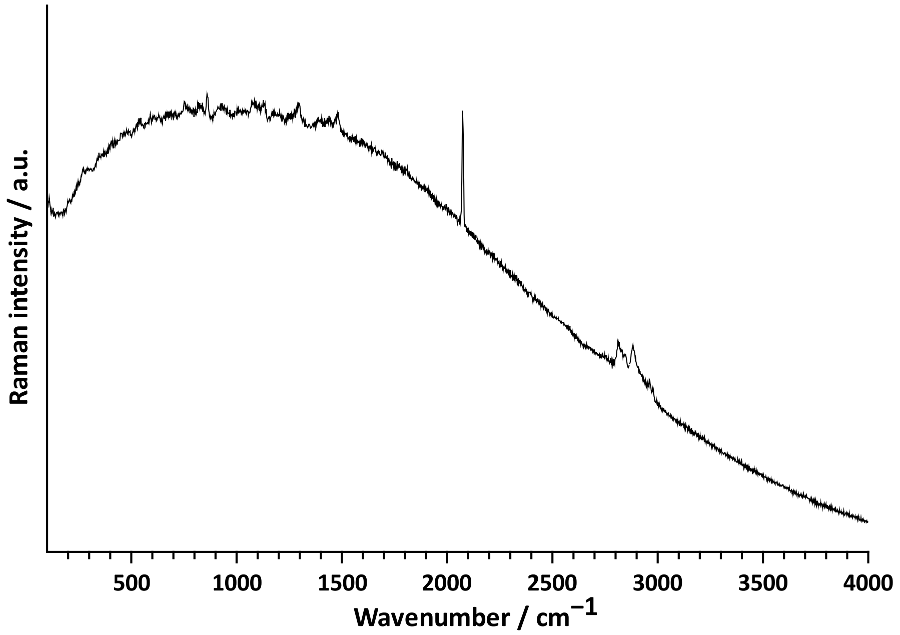

Figure S 15: Raman spectrum [K@crypt-222][TeCN] measured at room temperature without baseline correction.

2. Crystallographic data

Single-crystal X-ray diffraction data were collected using an IPDS-2T (Stoe, Darmstadt) diffraction system equipped with mirror monochromated Mo Kα radiation (λ = 0.7107 Å, Xenocs Microfocus Source) and an Image Plate detector. Crystals were selected under Paratone-N oil, mounted on micromount loops, and quench-cooled using an Oxford Cryosystems open-flow N_2 cooling device. Data were collected at 100 K and processed using the X-Area program package, including unit-cell parameter refinement and interframe scaling (which was carried out using LANA within X-Area). The structure was subsequently solved using direct dual-space methods (SHELXT)[2] and refined on F^2 with SHELXL[3] using the Olex2[4] user interface. The crystal structure drawings were generated with DIAMOND.[5] Single crystals suitable for single crystal X-ray diffraction were grown by diffusion of *n*-hexane in filtered solution of the titled compound in acetone. *Note:* In our experiment overlaying a conc. solution of the discussed compound in acetone with *n*-hexane led to precipitation of significant amounts of elemental tellurium (Figure S 16), however, colorless single crystals were obtained.

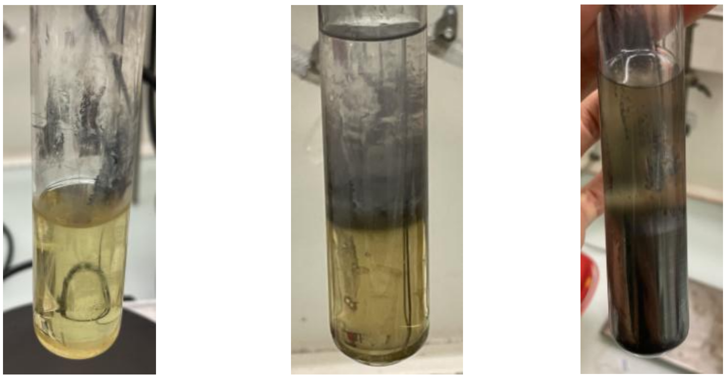

Figure S 16: [K@crypt-222][TeCN] acetone solution after filtration and attempt to concentrate (left). [K@crypt-222][TeCN] acetone solution freshly layered with *n*-hexane (middle). [K@crypt-222][TeCN] acetone solution layered with *n*-hexane after 3 days (right).

2.1 Single crystal X-ray data collection and refinement parameters

Table S 1: Selected single crystal X-ray data collection and refinement parameters for [K@crypt-222][TeCN].

	[K@crypt-222][TeCN]
Formula	C19H35KN3O6
CCDC	2428067
F. w. / g mol^{-1}	568.208
Crystal system	monoclinic
Space group	$P2_1/c$
a / Å	11.7362(5)
b / Å	14.3520(5)
c / Å	14.9300(7)
α / °	90
β / °	92.161(4)
γ / °	90
V / Å^3	2512.99(18)
Z	4
Radiation, λ / Å	MoKα (λ = 0.71073)
Temp / K	100
ρ_{calc} / g cm^{-3}	1.502
μ / mm^{-1}	1.385
Reflections collected	37988
Ind. Reflns. / Ind. Reflns gt	5777 / 2596
Parameters	271
R_{int} / $R_{(\sigma)}$ / %	0.0774 / 0.0509
$R1/wR2$,[a] $I \geq 2\sigma I$ / %	0.0453 / 0.1034
$R1/wR2$,[a] all data / %	0.0685 / 0.1159
GOF	1.0314

[a] $R1 = [\Sigma||F_o| - |F_c||]/\Sigma|F_o|$; $wR2 = \{[\Sigma w[(F_o)^2 - (F_c)^2]^2]/[\Sigma w(F_o^2)^2\}^{1/2}$; $w = [\sigma^2(F_o)^2 + (AP)^2 + BP]^{-1}$, where $P = [(F_o)^2 + 2(F_c)^2]/3$ and the A and B values are 0.055372 and 7.317125.

2.2 Additional Pictures of [K@crypt-222][TeCN]

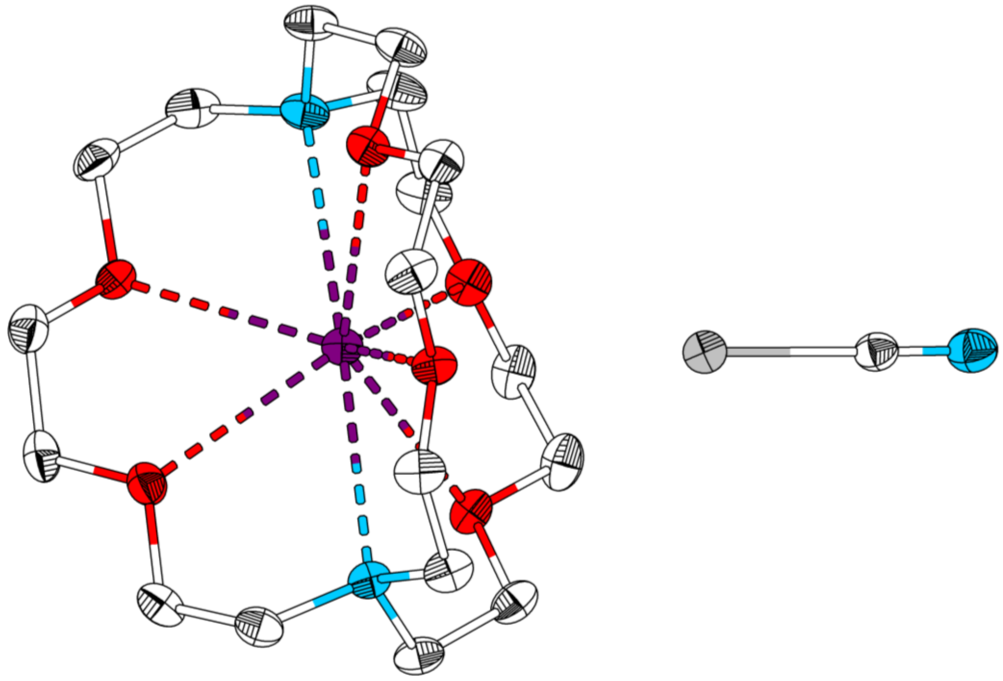

Figure S 17: Molecular structure of [K@crypt-222][TeCN] in the single crystal drawn with 50% displacement ellipsoids at 100 K. Hydrogen atoms are omitted for clarity. Colour code: K purple C white, N blue, O red and Te grey.

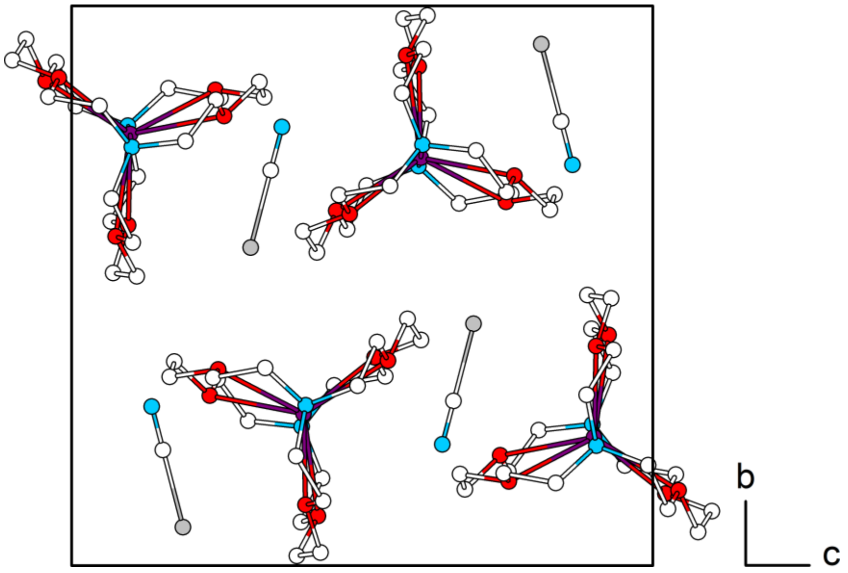

Figure S 18: Crystal structure of [K@crypt-222][TeCN] viewed along [100]. Atoms drawn with arbitrary radii. Hydrogen atoms are omitted for clarity. Colour code: K purple C white, N blue, O red and Te grey.

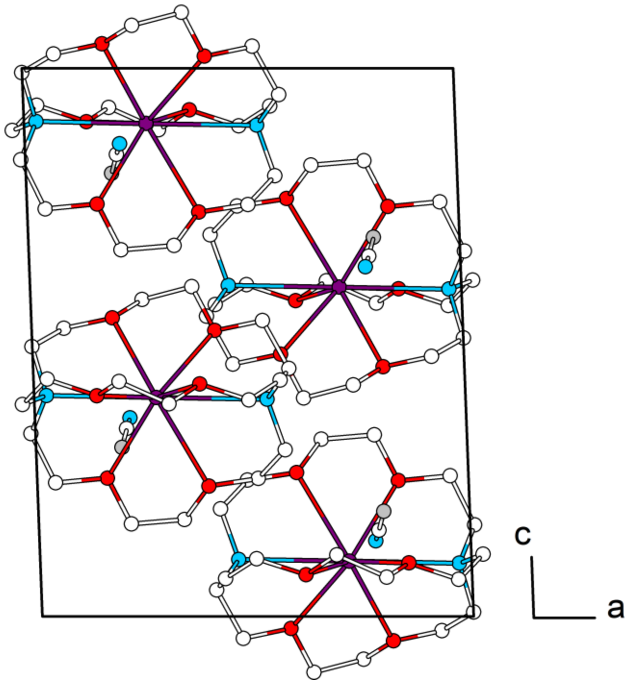

Figure S 19: Crystal structure of [K@crypt-222][TeCN] viewed along [010]. Atoms drawn with arbitrary radii. Hydrogen atoms are omitted for clarity. Colour code: K purple C white, N blue, O red and Te grey.

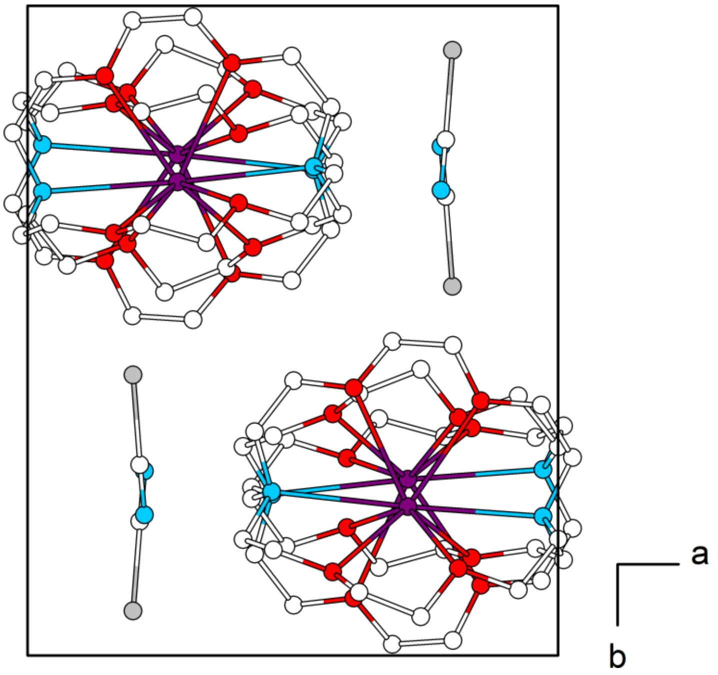

Figure S 20: Crystal structure of [K@crypt-222][TeCN] viewed along [001]. Atoms drawn with arbitrary radii. Hydrogen atoms are omitted for clarity. Colour code: K purple C white, N blue, O red and Te grey.

3. Comparison of $[TeCN]^-$ with comparable $[\mathit{Ch}CN]^-$ in literature

For comparison of $[TeCN]^-$ with its lighter homologues, we choose single crystal data of cyanate, thiocyanate and selenocyanate compounds with cations in which only weak van der Waals interactions are present. The bond C–N and C–*Ch* with *Ch* = O, S, Se are listed with their respective literature in Table S 2.

Table S 2: Bond lengths of selected cyanate, thiocyanate and selenocyanate with weak coordinating counter cations.

Bond lengths [Å]	$[^nPr_4N][OCN][CBr_4]$[6]	$[PPh_4][SCN]\ [C_2I_4]$[7]	$[^nPr_4N][SeCN]$[8]
C–N	1.145(4)	1.189(4)	1.153(2)
Ch–C	1.245(4)	1.675(3)	1.814(2)

4. Details on quantum chemical calculations

Calculations for chemical bonding analysis, NMR shielding and NMR coupling tensors were done with the program system TURBOMOLE[9] with all-electron relativistic methods at one- and at two-component level[10–15], with corresponding basis sets of triple zeta valence quality.[16] The PBE0 functional[17] was used, the conductor-like screening model in its variant employing Gaussian charge distributions[18] was employed with default parameters. The resulting NMR shifts (underlying data for Figure 5) are listed in Table S3, the coupling constants in Table S5. For the N shifts, experimentally NH_3 was used as reference. The choice of NH_3 as reference in calculations lead to a systematic shift of ~20 ppm, most probably due to intermolecular interactions between NH_3 molecules. This was corrected by using $(NH_3)_3$ instead and averaging over the three N atoms. The coordinates of $(NH_3)_3$ are given in Table S 4. They were generated by random distribution of three NH_3 molecules and subsequent optimization at level PBE[19]/x2c-TZVPall/D3[20]. This procedure was repeated ten times always leading to very similar structures with energies differing by only 6 kJ/mol and averaged NMR shielding constants agreeing within 1 ppm. We note in passing that the analogous procedure for the dimer leads to similar averaged shielding constants. They differ by ~4 ppm from those for the trimers. Canonical valence MOs of $(ECN)^{-}$ for E=O, Se are shown in Figure S22.

Table S 3: Calculated ^{13}C and ^{15}N NMR shifts in $[ECN]^{-}$ (E=O, S, Se, Te), in ppm, at two-component level (column "2c"), resolved to the contributions from unperturbed and B-field perturbed density (ρ, ρ^{B}) as well as corresponding spin-orbit contributions (ρ_{SO}, ρ^{B}_{SO}). Furthermore, scalar relativistic numbers (*i.e.*, without spin-orbit coupling), SR, and nonrelativistic numbers, NR.

	^{13}C								^{15}N							
E	ρ	ρ_{SO}	ρ^{B}	ρ^{B}_{SO}	2c	Expt	SR	NR	ρ	ρ_{SO}	ρ^{B}	ρ^{B}_{SO}	2c	expt	SR	NR
O	-16.2	0.1	150.6	-0.4	134.1	128	134.4	134.5	-12.7	0.3	86.0	-0.2	73.4	77	73.3	73.4
S	-12.3	0.1	154.9	-3.5	139.2	134	142.6	142.6	-11.5	0.2	227.6	-0.3	215.9	212	216.1	216.1
Se	-15.1	0.1	161.1	-21.9	124.2	121	146.0	144.3	-11.0	0.1	263.4	-1.1	251.4	241	252.3	252.6
Te	-16.5	0.0	164.2	-64.9	82.8	77	147.7	143.0	-12.9	0.0	307.9	-4.1	291.0	286	294.8	296.5

Table S4. Cartesian coordinates of $(NH_3)_3$, in Å, used as reference in the calculations of the chemical shifts.

N	0.0296619	-0.6440419	1.5793001
H	-0.7465554	-0.9869236	2.1511664
H	-0.0125198	-1.1437738	0.6858607
H	0.8952127	-0.9360949	2.0405132
N	-0.0078850	-1.1043415	-1.6840814
H	-0.8576369	-1.5327993	-1.3026939
H	0.7749113	-1.6459753	-1.3034973
H	0.0567610	-0.1760472	-1.2505576
N	0.2076869	1.8663947	-0.1192546
H	0.1290711	1.0555170	0.5243403
H	-0.6059945	1.8266150	-0.7414235
H	1.0244388	1.6909325	-0.7131937

Table S 5: Coupling constants in Hz calculated with and without SOC; for the latter also by accounting for the Fermi-contact (FC) term only.

	With SOC			Without SOC			Without SOC, FC only		
	Ch–C	C–N	Ch–N	Ch–C	C–N	Ch–N	Ch–C	C–N	Ch–N
$[^{17}OCN]^-$	37	11	2	37	11	2	28	7	6
$[^{33}SCN]^-$	41	10	2	42	10	3	36	5	4
$[^{77}SeCN]^-$	352	7	21	364	7	23	336	1	22
$[^{125}TeCN]^-$	911	6	58	998	5	72	937	0	57
$[^{125}TeCN]^-$ (exp.)	748	8	55	—	—	—	—	—	—

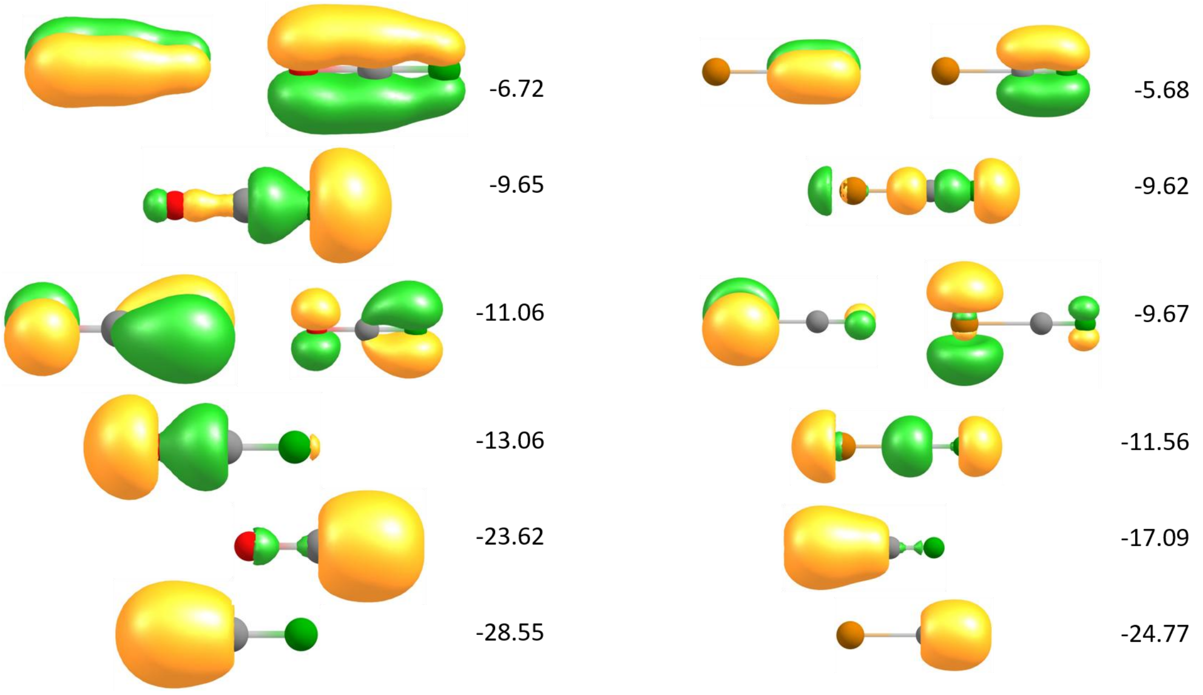

Figure S 21: Canonical valence MOs of $[OCN]^-$ and $[TeCN]^-$ and their energies (in eV) contours are drawn at ±0.08 a.u.

5. Negative ion photoelectron spectroscopy (NIPES) of [K@crypt-222][TeCN]

5.1 Experimental methods

The negative ion photoelectron spectroscopy (NIPES) was carried out using a magnetic-bottle time-of-flight (TOF) photoelectron spectrometer combined with a homemade electrospray ionization (ESI) source and a temperature controlled cryogenic ion trap.[21] The generated $[TeCN]^-$ anions from spraying a ~0.1mM [K@crypt-222][TeCN] solution dissolved in anhydrous acetonitrile were transported by a quadruple ion guide into a temperature controlled 3D ion trap, where they were accumulated for 20–100 ms and cooled by collisions with a cold buffer gas (20% hydrogen balanced in helium) to 20 K. The $[TeCN]^-$ anions were then pulsed out into the extraction zone of a TOF mass spectrometer at a repetition rate of 10 Hz, mass-selected, and decelerated before being photodetached by the laser from a tuneable OPO laser (Spectra-Physics ULD500) that was pumped by the 3rd harmonic of a Nd:YAG laser (Spectra-Physics Quanta-Ray Pro270). The probe laser was operated at a 20 Hz repetition rate with the anion beam shut off on alternating laser shots to afford real-time shot-to-shot background subtraction. The resulting photoelectrons were collected at nearly 100% efficiency in the magnetic bottle and analyzed with a 5.2 m long electron flight tube. Recorded flight times were converted into calibrated kinetic energies. Electron binding energies (EBEs) were obtained by subtracting the electron kinetic energies from the detachment photon energy with an energy resolution ($\Delta E/E$) of about 2% (*i.e.*, ~20 meV for 1 eV kinetic energy electrons).

5.2 Theoretical methods

Firstly, geometries of both $[TeCN]^-$ anion and corresponding neutral molecule are optimized with the CCSD(T) method coupled with aug-cc-pVTZ basis set[22] (aug-cc-pVTZ-PP for the Te atom[23]), followed by frequency analyses for both anion and neutral radical at the same level of theory to compute detailed vibrational frequencies and to afford zero-point energy

(ZPE) corrections. The electron affinity (EA) of [TeCN]$^{\bullet}$ was then calculated based on CCSD(T) energy difference (combined electronic energies and ZPE corrections) between neutral and anionic states. The spin-orbit coupling (SOC) split in the [TeCN] molecule were also calculated by setting up and diagonalizing spin-orbit matrices between non-interacting, spin-orbit-free basis states, which were obtained by CASPT2 calculations with CASSCF reference wave functions. The full valence active space composed of 15 electrons and 12 orbitals and all-electron basis sets (aug-cc-pVTZ-DK3 for the Te atom[24]) were used for the SOC calculations. All these calculations were carried out with the ORCA 5.0.4 program.[25,26]

6. References

[1] W. L. F. Armarego, C. L. L. Chai, *Purification of Organic Chemicals*, **2009**.
[2] G. M. Sheldrick, *Acta Crystallogr. Sect. Found. Adv.* **2015**, *71*, 3–8.
[3] G. M. Sheldrick, *Acta Crystallogr. A* **2008**, *64*, 112–122.
[4] O. V. Dolomanov, L. J. Bourhis, R. J. Gildea, J. A. K. Howard, H. Puschmann, *J. Appl. Crystallogr.* **2009**, *42*, 339–341.
[5] Brandenburg, K., Putz, H., *DIAMOND, Program for X-Ray Structure Analysis*, Crystal Impact GbR, Bonn, Germany, **1999**.
[6] S. V. Rosokha, C. L. Stern, A. Swartz, R. Stewart, *Phys Chem Chem Phys* **2014**, *16*, 12968–12979.
[7] H. Bock, S. Holl, *Z Naturforsch B* **2002**, *57*, 843.
[8] Nuzzo, Stefano, Twamley, Brendan, Baker, Robert. J., **2023**, DOI 10.5517/CCDC.CSD.CC2GVF81.
[9] "TURBOMOLE, Version 7.9 2024; a development of University of Karlsruhe and Forschungszentrum Karlsruhe GmbH 1989-2007, TURBOMOLE GmbH since 2007, available via https://www.turbomole.org.," **2024**.
[10] Y. J. Franzke, N. Middendorf, F. Weigend, *J. Chem. Phys.* **2018**, *148*, 104110.
[11] Y. J. Franzke, F. Weigend, *J. Chem. Theory Comput.* **2019**, *15*, 1028–1043.
[12] Y. J. Franzke, *J. Chem. Theory Comput.* **2023**, *19*, 2010–2028.
[13] Y. J. Franzke, C. Holzer, *J. Chem. Phys.* **2023**, *159*, 184102.
[14] Y. J. Franzke, F. Mack, F. Weigend, *J. Chem. Theory Comput.* **2021**, *17*, 3974–3994.
[15] K. Reiter, F. Mack, F. Weigend, *J. Chem. Theory Comput.* **2018**, *14*, 191–197.
[16] P. Pollak, F. Weigend, *J. Chem. Theory Comput.* **2017**, *13*, 3696–3705.
[17] J. P. Perdew, M. Ernzerhof, K. Burke, *J. Chem. Phys.* **1996**, *105*, 9982–9985.
[18] A. Pausch, *J. Chem. Theory Comput.* **2024**, *20*, 3169–3183.
[19] J. P. Perdew, K. Burke, M. Ernzerhof, *Phys. Rev. Lett.* **1996**, *77*, 3865–3868.
[20] S. Grimme, J. Antony, S. Ehrlich, H. Krieg, *J. Chem. Phys.* **2010**, *132*, 154104.
[21] Q. Yuan, W. Cao, X.-B. Wang, *Int. Rev. Phys. Chem.* **2020**, *39*, 83–108.
[22] T. H. Dunning, *J. Chem. Phys.* **1989**, *90*, 1007–1023.
[23] K. A. Peterson, D. Figgen, E. Goll, H. Stoll, M. Dolg, *J. Chem. Phys.* **2003**, *119*, 11113–11123.
[24] D. H. Bross, K. A. Peterson, *Theor. Chem. Acc.* **2014**, *133*, 1434.
[25] F. Neese, *WIREs Comput. Mol. Sci.* **2012**, *2*, 73–78.
[26] F. Neese, *WIREs Comput. Mol. Sci.* **2022**, *12*, e1606.
